# A Study of CRISPR Ribonucleoprotein Displacement in Cell-Free Systems

**DOI:** 10.1021/acsomega.4c09275

**Published:** 2025-02-26

**Authors:** Randi L. Smith, Peter W. Davenport, Matthew R. Lakin

**Affiliations:** † Center for Biomedical Engineering, University of New Mexico, Albuquerque, New Mexico 87131, United States; ‡ Department of Computer Science, University of New Mexico, Albuquerque, New Mexico 87131, United States; § Department of Chemical & Biological Engineering, University of New Mexico, Albuquerque, New Mexico 87131, United States

## Abstract

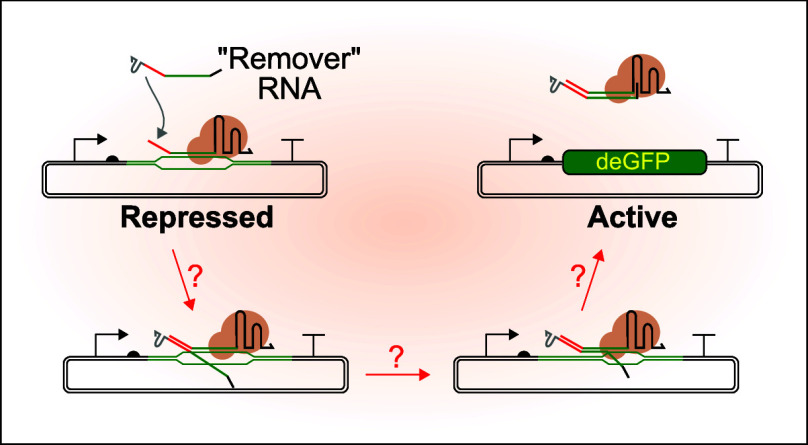

CRISPR/Cas-based transcription factors are a powerful tool for controlling gene expression in living cells and cell-free systems, as their programmable DNA-binding activity makes them a powerful tool for building and scaling up engineered genetic networks. The use of guide RNAs for targeting Cas proteins to desired binding sites opens up the possibility of using RNA engineering techniques to achieve programmable and dynamic control of CRISPR/Cas-based transcription factor activity and hence of gene expression. In this work, we investigate the use of RNA strand displacement systems to remove bound CRISPR/Cas ribonucleoprotein complexes from target DNA in cell-free systems. The binding of catalytically inactive dCas9 is monitored by using CRISPR interference to repress the expression of a reporter protein. We express an antisense RNA complementary to an extended toehold on an engineered guide RNA in an *E. coli*-based cell-free expression system with the goal of rapidly removing bound CRISPR/Cas ribonucleoproteins via strand displacement. We find that dCas9 appears to be surprisingly resistant to removal via this mechanism, which indicates that other strategies for dynamic removal of bound Cas proteins may prove to be more effective.

## 1 Introduction

The field of synthetic biology utilizes engineered, bioinspired methods to achieve programmable control over the behavior of cells and cell-like systems.^1,2^ The practical applications of achieving such control are wide-ranging, including the biosynthesis of valuable chemical products including biopharmaceuticals,^3^ the development of engineered bacteria for biomedical sensing^4,5^ or therapy,^6,7^ and the engineering of rationally designed abiotic “synthetic cells”.^8^ These applications require engineered mechanisms to control gene expression and a variety of tools has been developed to meet this need, including repurposed protein transcription factors from biology,^9^ RNA-based regulators of transcription^10,11^ and translation,^12^ and CRISPR/Cas-based transcription factor systems.^13,14^ The latter is the subject of the work outlined in this paper.

CRISPR-Cas is an RNA-guided endonuclease that forms an adaptive immune system against bacteriophage infection in a wide range of bacteria and archaea.^15^ Fragments of phage genomes are excised and inserted into a CRISPR array, which when expressed and processed forms a guide RNA which contains a spacer sequence complementary to part of the phage genomes and a conserved sequence for binding to a Cas protein. While a multitude of CRISPR-Cas systems have been discovered in recent years, the type II system from *Streptococcus pyogenes* is perhaps the most widely used, as only a single protein (Cas9) is required for DNA binding, in conjunction with a single guide RNA (sgRNA) that can be created by fusing the naturally occurring crRNA and tracrRNA sequences. Wild-type Cas9 is commonly used for genome editing and gene therapy applications thanks to its double strand cleavage activity.^16^ However, two point mutations suffice to deactivate both nuclease lobes and produce dCas9, a catalytically inactive variant that can be used as a programmable transcription factor thanks to its targeted DNA binding, but not cleavage, activity. The most straightforward use of CRISPR/Cas-based systems as transcription factors is CRISPR interference (CRISPRi), where the dCas9 is guided to a target promoter region or open reading frame and represses gene expression by blocking transcription initiation or elongation, respectively.^13^ This approach has also been used to build genetic logic circuits in yeast comprising NOT and NOR logic gates that repress the expression of downstream guide RNAs.^17^ Other uses of CRISPR for transcriptional control include CRISPR activation (CRISPRa), in which a transcriptional activator is recruited to a promoter region, for example via an RNA aptamer domain that is appended to the guide RNA.^14,18^ While CRISPR is clearly a powerful tool for controlling gene expression, unlocking its full potential requires developing mechanisms for dynamic control of CRISPR binding in genetic networks.

A number of techniques have been developed for control of CRISPR activity, including optically activated Cas9 and ligand-sensitive split Cas9 systems,^19^ as well as approaches that involve the expression of anti-CRISPR proteins isolated from biology^20^ and optically induced degradation of engineered photocleavable guide RNAs.^21^ Here, however, our primary focus is on RNA engineering approaches that use rational design of RNA–RNA interactions to control guide RNA structure, and thus function. Much previous work in this direction has focused on the use of hairpin-structured guide RNAs which are inactive in their folded state until they interact with a complementary RNA,^22,23^ including recent work on multi-input logical control.^24^ A similar approach has also been used to control the activity of Cas12a, an alternative Cas protein from *Acidaminococcus* that has a particularly compact sgRNA structure. Earlier work expressed a linear antisense RNA to block binding of dCas9 to its target;^25^ that work found that disrupting the structure of the Cas9 binding handle was the most effective means of blocking CRISPRi function; presumably by reducing the affinity of dCas9 for the sgRNA. In other recent work, a similar approach utilizing heavily chemically modified oligonucleotides was also able to block the binding of Cas proteins to target DNA.^26^

All of the above approaches, however, focused primarily on controlling the activity of the Cas protein prior to its interaction with the DNA, mostly by relieving some secondary structure that was preventing the RNA-DNA interaction. This led us to consider whether DNA strand displacement might offer a means of removing a bound CRISPR ribonucleoprotein (RNP) complex *after* it had bound to target DNA. Toehold-mediated strand displacement has developed into a powerful tool for programming the dynamics of DNA nanostructures and molecular computing devices.^27−29^ Additional work has established similar RNA strand displacement reactions in various contexts^30−32^ and RNA strand displacement across RNA and RNA:DNA hybrids has also been reported.^33,34^ In an attempt to achieve more precise control over the timing of CRISPR RNP binding and removal reactions, we chose to carry out these reactions in an *in vitro* cell-free transcription and translation (TXTL) system derived from crude extracts of *E. coli*.^35^ Cell-free TXTL allows straightforward addition of new components to the reaction volume after the reaction has started and this system has previously been demonstrated to support expression of CRISPR proteins and CRISPR interference reactions.^36^ Developing a system for removal of bound CRISPR transcription factors would provide a powerful capability for dynamically controlling gene expression in cell-free systems, which has hitherto been challenging, although impressive work on more complex incoherent feedforward loops has shown success.^37^ In this study, however, our results do not provide strong evidence either for our against the displacement reaction occurring in our system.

## 2 Results

### 2.1 System Design

**Figure 1:**
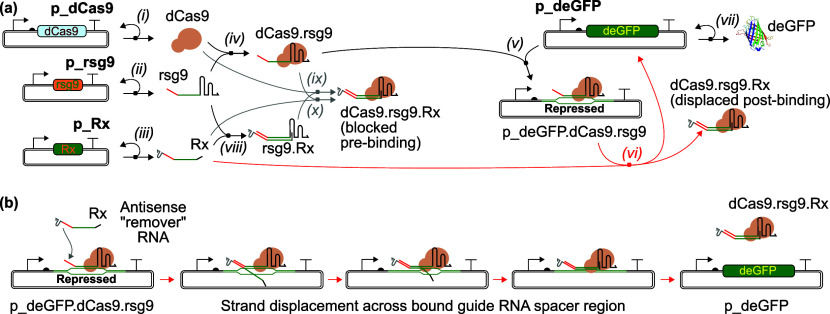
System outline for control of CRISPR interference via displacement of bound CRISPR ribonucleoproteins (RNPs). (a) Scheme of proposed reactions in the cell-free TXTL system. Relative plasmid concentrations in our experiments: [p_deGFP] = 1× , [p_dCas9] = 3× , [p_rsg9] = variable, [p_Rx] = variable. Here, and elsewhere, we use the notation “p_X” for the plasmid that expresses product X. (b) Detailed scheme for proposed displacement-mediated removal of a CRISPR RNP from target DNA (reaction *vi* from part (a)) via RNA strand displacement across the RNA:DNA hybrid region where the spacer binds to its target.

The scheme for our experimental system is outlined in Figure 1a. Major system components are synthesized from plasmids: dCas9 (reaction *i* from that Figure), a removable sgRNA (rsg9) extended with a linear binding region to facilitate displacement (reaction *ii*), and an antisense “remover” RNA (reaction *iii*). The dCas9 protein can bind to the removable sgRNA to create an active RNP complex (reaction *iv*). This can then interact with the deGFP plasmid to block the deGFP open reading frame (reaction *v*). Critically, the remover RNA may bind to, and displace, the CRISPR RNP complex from the deGFP plasmid (reaction *vi*), thereby enabling deGFP expression to take place (reaction *vii*). Alternatively, the remover might bind to the sgRNA and/or dCas9 complex before it binds to the deGFP plasmid, and thereby inhibit its repressive activity (reactions *viii,ix,x*). Our proposed mechanism for removal via CRISPR RNP displacement across the bound spacer region of the guide, is presented in more detail in Figure 1b. Briefly, we hypothesize that, in the presence of complementary linear binding regions on the sgRNA and the antisense remover RNA, the remover may bind via the linear region and initiate a branch migration reaction across the DNA:RNA hybrid region that anchors the CRISPR RNP to its target, thus displacing it from the DNA. However, as noted above, there is also the possibility of antisense binding that blocks an RNP from binding to its target ahead of time. Distinguishing between the two possible modes of repression, pre hoc blocking (reactions *viii,ix,x*) and post hoc displacement-mediated removal (reaction *vi*) was therefore a key goal of the experiments presented below.

Inspired by previous work, we use deGFP as a fluorescent reporter protein, regulated by dCas9 guided by a guide RNA which targets a binding site partway along the deGFP coding sequence. This guide RNA (sg9) and the corresponding nontargeting control guide RNA (sgNT) were derived from sequences used in previous work from the literature.^36^ The guide RNA sequences were modified by the addition of a 5′ extension intended to serve as a binding site to nucleate a strand displacement reaction; we refer to these as “removable single guide RNAs” (rsgRNAs, e.g., rsg9). The sequences for the extension domains were designed computationally using NUPACK^38,39^ to minimize both the secondary structure of the extended region and its impact on the overall predicted secondary structure of the guide RNA. Plasmids to express corresponding antisense RNAs, which we termed “removers”, were designed to express the remover under a strong Anderson promoter (J23119). All experiments were carried out in a commercially available cell-free TXTL derived from *E. coli* cell extracts. We refer the reader to Section 4 for full details of reagents, plasmid sequence designs, cloning protocols, and experimental methods.

### 2.2 Initial Experiments with Removable Guide RNAs

**Figure 2:**
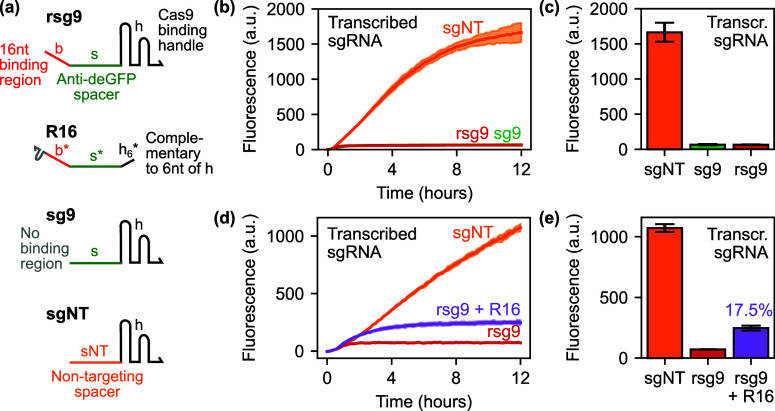
Initial characterization of removable guide RNAs. (a) RNA species used in the experiments presented in this figure. In these initial experiments, we used removable sgRNAs (rsgRNAs) with a 16 nt linear binding region; the corresponding complementary remover RNA is thus called R16. Here and henceforth, the on-target sgRNA used is called sg9 and the corresponding removable sgRNA is rsg9, following the nomenclature of Marshall et al.^36^ (b) Fluorescence timecourse data showing CRISPR interference performance of rsgRNA to the variant without the binding region, with sgRNAs transcribed from plasmids. The behavior of the two is indistinguishable, showing that rsg9 is functional for CRISPRi. Line shows mean of three replicates; shaded area is one standard deviation above and below the mean. (c) End point fluorescence data from the experiment shown in part (b). (d) Initial fluorescence timecourse data on removal of CRISPR RNPs by the R16 remover RNA. Modest recovery of the deGFP fluorescence signal is observed. Line shows mean of three replicates; shaded area is one standard deviation above and below the mean. (e) End point fluorescence data from the experiment shown in part (d). For the condition with R16 present, the annotation shows the percentage removal of bound dCas9 achieved as estimated via linear interpolation between the mean fluorescence values from the negative and positive controls.

We began by characterizing a removable sgRNA system consisting of an rsgRNA (rsg9) with a 16 nucleotide (nt) linear binding region; the sgRNAs (including control RNAs) used in these experiments are illustrated in Figure 2a. The sg9 and sgNT sequences were derived from the literature;^36^ the rsg9 removable sgRNA sequence was created by using NUPACK^40^ to design a 16 nt linear binding region with minimal secondary structure and minimal impact on the predicted fold of the core sgRNA sequence. This sequence was then used to design the complementary remover with a 16 nt complementary linear binding region, here termed “R16”. We first studied the CRISPR interference (CRISPRi) functionality of the rsg9 guide RNA in cell-free TXTL: the three sgRNAs were transcribed from plasmids in a one-pot reaction in the presence of additional plasmids expressing the deGFP reporter protein and the dCas9 protein required for CRISPRi. The observed fluorescent signal (Figure 2b,c) was indistinguishable between the sg9 and extended rsg9 sgRNAs, confirming that that the addition of the linear binding region did not interfere with the CRISPRi activity.

We next carried out experiments with ssDNA oligonucleotide mimics of the R16 remover in large excess, as the use of ssDNA allowed us to easily supply greater quantities of the remover than would be possible via *in situ* transcription within the cell-free system. Our results from this experiment showed no increase in the fluorescent signal at all in the presence of a large excess of ssDNA remover mimics (Figure S1). We hypothesized that the excess of ssDNA in the system might be inhibiting protein synthesis, however, we investigated the effect of these ssDNAs on deGFP expression alone and found no effect (Figure S2). We therefore concluded that ssDNA oligonucleotides may not be a good analog for antisense RNAs and thus did not investigate these further.

We then introduced an additional plasmid expressing the R16 antisense remover RNA, and observed the CRISPRi results from the rsg9-expressing plasmid with and without the remover plasmid being expressed. (An additional “filler” plasmid was added in all cases to control the total amount of plasmid DNA across all conditions.) We observed a modest recovery in fluorescent signal (Figure 2d,e) which could be attributed either to displacement-mediated removal of the CRISPR RNPs or to pre hoc antisense blocking of the RNPs. Additional data from a second characterization experiment, which showed similar results, is presented in Figure S3. These results provided some evidence for the displacement-mediated removal of CRISPR RNPs and paved the way to our subsequent studies of this system.

### 2.3 Further Investigation of Plasmid-Expressed Removable Guide RNAs

**Figure 3:**
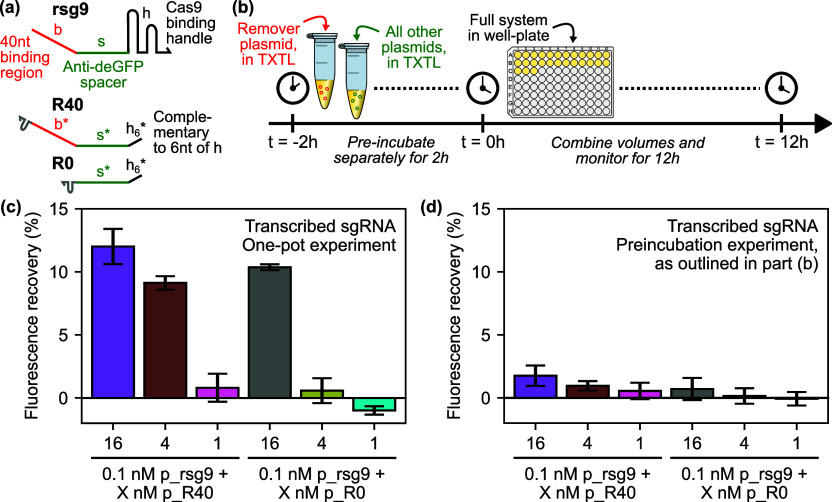
Further investigation of remover RNA activity against coexpressed and preincubated removable sgRNAs. (a) New RNA species used in the experiments presented in this figure. Here and henceforth, we used removable sgRNAs (rsgRNAs) with a 40 nt linear binding region; the corresponding complementary remover RNA is thus called R40. (b) Experiment outline for preincubation of CRISPR RNPs with target DNA prior to the addition of plasmid-expressed remover RNA that has been separately incubated for the first 2 h of the experiment. (c) End point data from a one-pot experiment, presented as percent fluorescence recovery, normalized between the off-target (sgNT) and on-target (rsg9 only) controls. Modest fluorescence recovery is again observed. Negative fluorescence recovery percentages indicate lower end point fluorescence than the rsg9 positive control, which could be attributed to load on the TXTL system. Bars show means of three replicates; error bars are one standard deviation. Note that the *y*-axes of this and similar subsequent plots have been chosen to provide maximal clarity on the fluorescence recovery values across their relevant ranges. (d) End point data from a preincubation experiment, presented as percent fluorescence recovery. Significantly lower fluorescence recovery is observed, which suggests difficulty displacing the preincubated, transcribed rsgRNAs from their targets. Bars show means of three replicates; error bars are one standard deviation. Unprocessed end point data corresponding to this figure is presented in Figure S5.

We hypothesized that the fluorescence response observed in Figure 2d,e might have been limited by the length of the linear binding region. While a 16 nt binding region is long by the standards of *in vitro* toehold-mediated DNA strand displacement reactions,^41^ which explains why we attempt here to avoid referring to these reactions as “toehold-mediated”, other work on RNA strand displacement in cell-free systems has used longer binding regions, for example, previous work on small transcription-activating RNAs (STARs).^10,11^ We therefore designed a variant of rsg9 with a longer 40 nt linear binding region, together with a corresponding complementary extended remover RNA (R40) and a control remover RNA without the linear region (R0), as illustrated in Figure 3a. Results from NUPACK analysis of the predicted minimum free energy folds of these RNAs, both individually and in remover:sgRNA complexes, are presented in Figures S13–S15, illustrating that the structures fold as anticipated and should be able to hybridize with the remover, at least in the absence of dCas9 and target DNA. This extended rsg9 and remover were used for all subsequent experiments.

In addition, we developed an alternative experimental protocol in an attempt to differentiate between the pre hoc “blocking” and post hoc “displacement-mediated removal” modes of anti-CRISPRi activity (reactions *vii,ix,x* or *vi* respectively from Figure 1). The rationale behind this modified protocol was that if the CRISPR RNPs were preincubated with target DNA in the absence of the remover RNAs, they would be able to bind to their target before being exposed to the remover and thus the “displacement” mode of removal should be more dominant. This was achieved by initially incubating the remover plasmid in TXTL separately from all other plasmids for 2 h, before combining into a single reaction volume for subsequent monitoring, as outlined in Figure 3b (see Section 4 for full details).

These extended rsgRNA and remover RNAs were characterized experimentally, using both the one-pot and preincubated reaction protocols (Figure 3c,d). In these experiments, the concentration of the remover-expressing plasmid was titrated from 16 nM down to 1 nM and the remover variants with (R40) and without (R0) the 40 nt linear binding region complementary to the binding region on the rsgRNA were compared. In the one-pot experiment (Figure 3c), concentration-dependent recovery of the deGFP fluorescence signal was observed both the R40 and R0 removers, with maximum extent similar to that observed in the previous experiments. At the highest concentration (16 nM), the effects of the R40 and R0 removers were similar, which argues against a strand displacement mechanism. In addition, the fact that the highest concentration of the R0 remover produced a degree of signal recover indicates that there is a component of derepression seemingly not involving toehold-mediated strand displacement, possibly due to antisense binding of the remover to the guide RNA, which might delay or prevent formation of active ribonucleoprotein complexes. Strikingly, the dose–response curves of the two removers differ at the midrange around 4 nM, indicating that in this concentration range there may be an enhanced role for displacement-mediated action, although this could also be attributed to the longer length of the complementary region in this case providing a stronger antisense effect at this concentration. The preincubated version of this experiment Figure 3d was carried out in an attempt to differentiate these two modes of action, in particular for the R40 remover. Strikingly, the fluorescence recovery observed in this experiment was significantly lower than for the one-pot experiment, indicating that the CRISPR RNPs, once preincubated and bound, were not being efficiently removed from target DNA.

In the previous experiments, we used “filler” DNA plasmids to ensure that the total amount of DNA in the cell-free system was constant across all conditions; this is important because the presence of additional DNA titrates resources from the TXTL, such as RNA polymerases, away from the other plasmids in the system. To confirm that the identity of this filler DNA being used in our experiments was not contributing to the observed behavior, we tested the system using the commercially available pUC19 plasmid as the filler DNA (Figure S4); this experiment suggested that the filler DNA was not affecting the overall behavior of the system, although we note that plasmid crosstalk is a general problem that must be controlled for carefully in cell-free TXTL experiments.^42^

We also hypothesized that the length of the R40 remover RNA, which is complementary to not only the 40 nt linear binding region on the sgRNA and the 20 nt spacer region but also 6 nt of the conserved Cas9 binding handle, could be interfering with the ability of the remover to displace across the DNA:RNA hybrid region in this crowded space that contains both strands of the target DNA, the two RNA strands (rsgRNA and remover), and the dCas9 protein itself. Therefore, we tested truncated plasmid-expressed removers that remove 6, 12, or 18 nt of complementarity from the 5′ end of the remover, that is, from the distal end of the displacement domain. We hypothesized that even displacing a short region of the spacer domain could suffice to remove the RNP from its target, as 5′ truncations on an sgRNA should reduce the binding affinity for target DNA, as distal mismatches do.^43^ However, in our experiment these truncated removers performed less well than the original version (Figure S6), so we did not investigate these further.

### 2.4 Characterizing CRISPR Interference with Synthetic Guide RNAs

**Figure 4:**
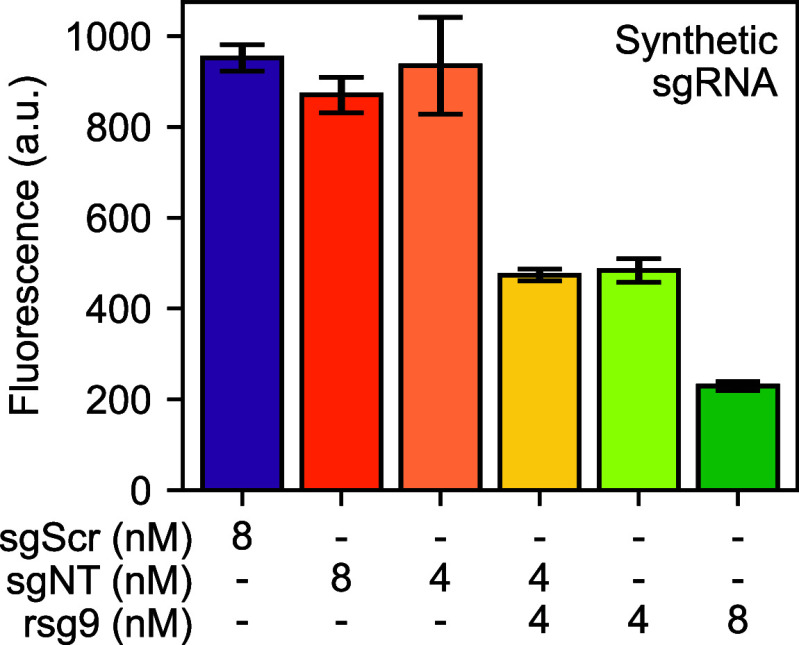
Characterization of synthetic sgRNA in CRISPR interference reactions. Plasmids expressing deGFP and dCas9 were tested with the concentration of the corresponding sgRNA shown in each case. As before, rsg9 is the on-target anti-deGFP removable sgRNA and sgNT is an off-target control. The “sgScr” control is a control RNA containing the same nucleotides as sgNT but in a scrambled order; it should thus not interact with dCas9 at all. The concentration of deGFP plasmids was 0.4 nM, so 4 nM of synthetic RNA corresponds to a 10-fold excess, and 8 nM corresponds to a 20-fold excess. Bars show means of three replicates; error bars are one standard deviation. We observed strong repression in the presence of 20-fold synthetic sgRNA, which we used in all subsequent experiments.

In cell-free systems, plasmid-encoded components are synthesized continually, typically at a constant rate until the resources of the TXTL start to become depleted. In the experiments outlined above, all components, including the rsgRNAs, were expressed from plasmids under constitutive promoters and thus were continually produced throughout the course of the experiment. This means that, over time, the amount of rsgRNA for the remover RNAs to interact with, increases over time but the quantity of the DNA target remains constant. Thus, we hypothesized that there might be so much rsgRNA in the system that remover RNAs were titrated away from CRISPR RNPs by unbound rsgRNAs (reactions *viii,ix,x* from Figure 1) and thus be less likely to displace RNPs already bound to target DNA. Therefore, we began to test the use of synthetic rsgRNAs in our cell-free systems in place of plasmid-expressed rsgRNAs, as in case there is only a fixed supply of rsgRNA to interact with throughout the experiment. We began by characterizing the CRISPRi performance of synthetic sgRNAs and rsg9 as a function of the concentration of the synthetic RNA, in a simple one-pot experiment with no preincubation. The results of this experiment are shown in Figure 4. We found that 8 nM produced strong repression of deGFP expression and that the results were linear with concentration of the on-target guide RNA. We therefore used 8 nM concentrations of synthetic sgRNAs in all subsequent experiments.

### 2.5 First Study of Strand Displacement-Mediated Removal of Synthetic Guide RNAs

**Figure 5:**
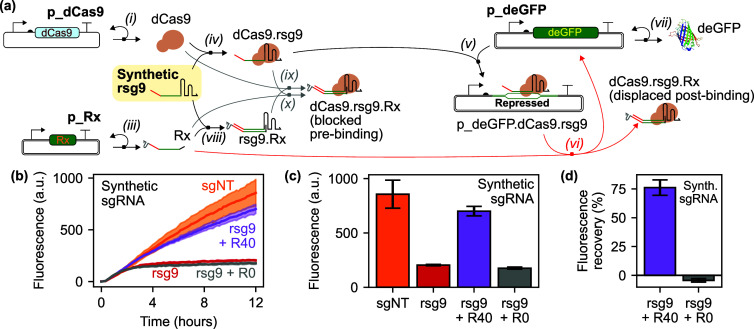
Initial study of removal of CRISPR RNPs using synthetic sgRNAs, in a one-pot experiment. (a) Scheme of proposed reactions in the cell-free TXTL system when synthetic sgRNAs are used. The only difference is that a finite quantity of the synthetic sgRNA is provided at the start, rather than it being synthesized continually from a plasmid during the experiment. We hypothesized that using a fixed supply of sgRNA would make it easier for our antisense remover RNAs to operate, as there would be less sgRNA to remove overall. (b) Fluorescence timecourse data showing performance of removers against synthetic sgRNAs. The R40 and R0 removers are as outlined in Figure 3a. The observed recovery of the deGFP fluorescence signal in the presence of R40 (remover with linear binding region) could indicate displacement-mediated removal of CRISPR RNPs, as no such signal is seem with R0 (control remover without linear binding region). Line shows mean of three replicates; shaded area is one standard deviation above and below the mean. (c) End point fluorescence data from the experiment shown in part (b). (d) End point fluorescence data from part (c), expressed as percentage fluorescence recovery by normalizing between the rsg9 negative control and sgNT positive control.

Having validated the CRISPRi functionality of synthetic rsgRNAs, we tested those rsgRNAs in experiments with antisense removers as above. The proposed scheme for these reactions is outlined in Figure 5a, which is similar to the scheme from Figure 1a but with *ii* removed, as the rsgRNA is no longer being synthesized from a plasmid. We carried out this initial characterization in a one-pot reaction and compared the responses of the R40 and R0 removers, which were expressed from a plasmid as before. Results are presented as a fluorescence timecourse Figure 5b, end point fluorescence Figure 5c, and interpolated percentage fluorescence recovery Figure 5d. Here, the R0 remover produced almost no fluorescence recovery whereas the R40 remover (with the complementary linear binding region) produced the strongest fluorescence recovery seen so far, around 75%. The results observed for R0 are consistent with there being high initial concentrations of rsg9 RNA, therefore greater quantities of R0 would be required early in the reaction in order to delay dCas9:rsg9 complex formation. These data also suggest that derepression of deGFP expression previously observed in the presence of the R0 remover strand is via pre hoc antisense-mediated blocking of dCas9:rsg9 complex formation. Therefore, R0 is ineffective at relieving GFP repression once such complexes have formed, whereas R40 may be able to displace rsg9 from dCas9:rsg9 complexes and so can continue to derepress throughout the reaction. This data is thus suggestive of post hoc anti-CRISPRi activity by R40 via displacement-mediated mode of removal of Cas, when the quantity of sgRNA in the system is limited to a level that allows the remover to operate effectively. However, subsequent experiments with additional controls and modified protocols were clearly needed to try to tease out the relative contributions of pre hoc blocking and post hoc displacement.

### 2.6 Studying Removal of Preincubated Synthetic Guide RNAs

**Figure 6:**
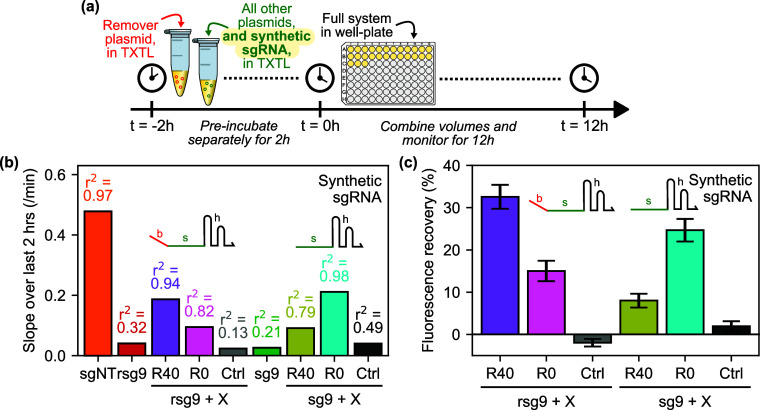
Studying removal of preincubated CRISPR RNPs using synthetic sgRNA. (a) Experiment outline for preincubation of CRISPR RNPs, including synthetic sgRNAs, with target DNA prior to the addition of plasmid-expressed remover RNA that has been separately incubated for the first 2 h of the experiment. (b) Results of linear regression to calculate rates of change of deGFP fluorescence over last 2 h of a preincubation experiment in the presence of removers with (R40) and without (R0) a 40 nt linear binding region, and a control RNA (Ctrl), for synthetic sgRNAs with (rsg9) and without (sg9) a complementary 40 nt linear binding region. Bars indicate slope of fluorescence timecourse over the final 2 h, with *r*^2^ values annotated. (c) End point fluorescence values from the same experiment shown in part (a), presented as percentage fluorescence recovery interpolated between the negative (sgNT) and positive (rsg9) controls. Bars are averages of three replicates; error bars show one standard deviation. See Figure S111 for raw timecourse and end point data, which were used to perform the regression analysis and calculate end point fluorescence recovery values presented here.

We next investigated the more challenging case of attempting to remove synthetic rsgRNAs that were preincubated with their targets prior to the addition of plasmid-expressed remover RNAs, as outlined in Figure 6a. Data from a preliminary experiment on that topic is presented in Figure S7; the fluorescence timecourse data (Figure S7b) and analysis of rates of reporter synthesis via linear regression (Figure S7c) demonstrated that the remover RNA (R40) produced a significantly stronger signal than the noninteracting control RNA (Ctrl). As expected, the fluorescent signal recovery was lower than that seen in Figure 5, as in that experiment the removers were not preincubated with their target and thus pre hoc blocking of CRISPR RNP binding plays a greater role. Data from additional experiments that are consistent with those shown here are presented in Figures S8 and S9.

Processed results from a more comprehensive experimental setup that tests *all* combinations of the R40 and R0 removers in conjunction with the removable rsg9 and the original version sg9 (without the linear binding region) are presented in Figure 6b,c. Here we see that the strongest response, in terms of rates of reporter synthesis over the last 2 h of the experiment, is observed in the case of the R40 remover and the rsg9 removable sgRNA; significantly stronger than the signal for R0 with rsg9. Interestingly, this behavioral trend was reversed when testing R40 and R0 with the nonremovable sg9 guide RNA, and with similar rates of reporter synthesis. These results could be interpreted as evidence for a displacement-mediated effect via the binding region of R40 and rsg9, given the lower response seen with R0 and rsg9, or simply a stronger antisense effect given the 40 additional nucleotides of complementarity between the remover RNA and the guide RNA in the former case. However, the signal for R0 and sg9 (which presumably incorporates no displacement-mediated component) is similar to that for R40 and rsg9, and higher than the signal for R40 and sg9 which has the same number of base-pairs of complementarity between the remover RNA and guide RNA. This argues against a displacement-mediated reaction: the commonality between the lower-fluorescence cases here is the fact that one RNA is 40 nt longer than the other, suggesting that the destabilizing effect of this overhang on the RNA–RNA interaction may be responsible for the lower signal in these cases. However, the timing of the onset of the rise in fluorescence (see Figure S11 for the raw fluorescence data) is reflected in the final fluorescence recovery values from Figure S11c; there we see that the fluorescence signal in the condition containing rsg9 and R40 is slightly stronger, suggesting that there might be a displacement-mediated mechanism at work that is speeding up the kinetics of CRISPR RNP removal. An additional experiment was carried out using this protocol whose results were similar but less equivocal, in particular in the case of the conditions containing rsg9 and either R40 or R0; those results are presented in Figure S10. The results for the sg9 conditions replicate those from Figure 6 quite well, suggesting that there may be experimental error in at least one of the rsg9 conditions.

### 2.7 Titration of Remover Plasmid Concentrations

**Figure 7:**
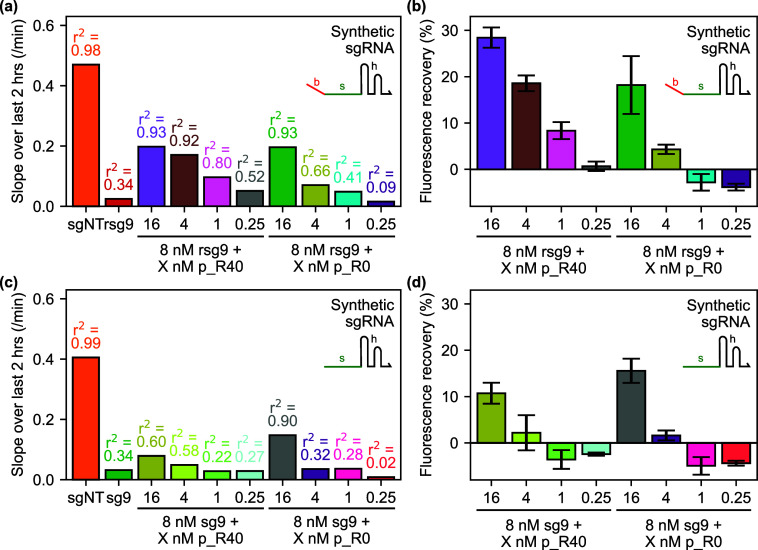
Remover plasmid titrations, for removal of preincubated synthetic sgRNA. (a) Results of linear regression to calculate rates of change of deGFP fluorescence over last 2 h of a preincubation experiment in the presence of removers with (R40) and without (R0) a 40 nt linear binding region, for synthetic sgRNAs with a complementary 40 nt linear binding region (rsg9). Bars indicate slope of fluorescence timecourse over the final 2 h, with *r*^2^ values annotated. (b) End point fluorescence values from the same experiment shown in part (a), presented as percentage fluorescence recovery interpolated between the negative (sgNT) and positive (rsg9) controls. Bars are averages of three replicates; error bars show one standard deviation. (c) Results of linear regression to calculate rates of change of deGFP fluorescence over last 2 h of a preincubation experiment in the presence of removers with (R40) and without (R0) a 40 nt linear binding region, and a control RNA (Ctrl), for synthetic sgRNAs without a complementary 40 nt linear binding region (sg9). (d) End point fluorescence values from the same experiment shown in part (c), presented as percentage fluorescence recovery interpolated between the negative (sgNT) and positive (sg9) controls. Bars are averages of three replicates; error bars show one standard deviation. Less fluorescence recovery was observed than in part (b), suggesting at least partial toehold-mediated removal of CRISPR RNPs is occurring. See Figure S12 for raw timecourse and end point data, which were used to perform the regression analysis and calculate end point fluorescence recovery values presented here.

Finally, we carried out a similar preincubation experiment with synthetic sgRNAs, this time titrating remover-expressing plasmid concentrations to determine the dose–response curve for both the R40 and R0 removers acting on the rsg9 and sg9 guide RNAs. Processed data from this experiment is presented in Figure 7. For both guide RNAs, we saw the strongest fluorescent signal at the highest concentration of each remover plasmid tested (16 nM). This is more consistent with the expected behavior than that seen in Figure 6, in that there was more obviously stronger fluorescence recovery in the condition containing both rsg9 and R40 than any other, and the conditions containing the control sg9 sgRNA saw significantly lower fluorescence recovery. At the second-highest concentration (4 nM), we observed a significantly stronger response in the condition with R40 and rsg9, and all lower concentrations showed significantly lower signals in all cases. Furthermore, while the final slopes of the fluorescence timecourses are similar in the cases of R40 and R0, the fluorescence timecourse (Figure S12a,c) show a delayed onset of fluorescence which could imply that the free guide RNAs are being saturated by the removers. As per Figure 5, the increased derepression of GFP by R40 relative to R0 is suggestive of post hoc anti-CRISPR activity. This interpretation is buttressed by the lower signal levels observed for rsg9 than for sg9 without the linear binding region (Figure 7c,d). However, our experimental design does not allow for unambiguous distinction between pre hoc antisense blocking and post hoc displacement-mediated activities. Indeed, the observed effect could be partially explained by the stronger antisense effect given the extended region of complementarity between rsg9 and R40.

## 3 Discussion

In summary, we have carried out a range of cell-free experiments to study the interactions of RNA strand displacement processes with CRISPR interference transcription factors, building toward the goal of displacement-mediated removal of bound CRISPR ribonucleoproteins (RNPs) from target DNA in cell-free transcription and translation (TXTL) systems. This is of practical interest given that DNA does not replicate in cell-free TXTL, which means that there are no replication forks helping to dislodge CRISPR RNPs from their targets. In principle, RNA–RNA interactions are a powerful tool for controlling CRISPR systems and have also been explored by other workers.^22−24,44^ Here, we have explored the utility of cell-free TXTL as a platform to study these interactions in some detail.

A key aspect of the work reported here is distinguishing between the two modes of derepression of a target gene: antisense blocking of binding of CRISPR RNPs to target DNA, and active displacement-mediated removal of CRISPR RNPs from their target DNA. In principle, the cell-free system offers a convenient platform for carrying out experiments with multistep protocols that could be used to tease out some of these differences. The challenge with such experiments is that, once a droplet of cell-free TXTL has been dispensed into a V-bottom well for reading in a plate reader, attempting to mix additional reagents into that droplet tends to disrupt the shape and position of the bubble which makes subsequent reads unreliable. Therefore, in this work we settled on a protocol in which initial components were mixed and incubated in a PCR tube in a thermal cycler for the initial incubation period, after which any additional components were added before the augmented mixture was pipetted into the well plate for subsequent monitoring in the plate reader. The disadvantage of this approach is that reporter expression cannot be monitored prior to the final mixing step. Nevertheless, this is a potentially valuable approach for experiments such as those presented in Figures 6, 7, and S7, where we wished to give the CRISPR RNPs the opportunity to form and prebind to their target DNA before attempting to remove them via addition of expressed complementary “remover” RNAs. In addition, the relative slowness of protein synthesis means that the preincubation experiment gives time for the dCas9 protein to be transcribed and translated, and to fold, mature, and bind to its guide RNA, before the remover RNA is added. In experiments such as that shown in Figure 5, there will be a window of time before mature dCas9 is available during which transcribed remover RNA will still be able to bind to the sgRNA, thereby blocking it from interacting with the target DNA at all.

As outlined above, there is still some difficulty in distinguishing between the two modes of derepression: antisense blocking vs active displacement-mediated removal of RNPs from their target DNA. It is likely that both mechanisms are operating in tandem, and the challenge is to determine their relative strengths. Data presented in Figure 7, as well as Figures S7–S9, appears suggestive of a toehold-mediated component in the derepression process, as evidenced by fluorescence recovery in the presence of remover RNAs carrying the complementary binding region. However, other results, such as those from Figures 6 and S10, are more equivocal. Our use of the multistep experimental protocol aimed to tease apart the effects by preincubating CRISPR RNPs with their target DNA ahead of the addition of complementary remover RNAs, and this showed that there is likely a combination of antisense blocking and displacement-mediated removal acting on the CRISPR ribonucleoprotein complexes. It is not clear why the level of derepression by R0 in Figures 6 and 7 is higher than that seen in Figure 5; further work may be needed to fully understand this observation. It is worth noting that our experiments were conducted using multiple batches of commercial cell extract, which might account for some inconsistencies observed in our results, but likely not all. Furthermore, RNA degradation is known to occur in cell-free systems,^45^ and this may contribute to the results seen here. For example, it may partially explain why higher concentrations were needed for synthetic sgRNA in the experiments shown in Figure 4 and subsequently.

Furthermore, in an attempt to highlight any effects from the displacement-mediated process, we switched to using synthetic sgRNAs in the CRISPR system instead of plasmid-expressed ones. The rationale here was to limit the total amount of sgRNA in the system rather than continually expressing more, so that there would be fewer unbound sgRNAs to sequester the plasmid-expressed remover RNAs. We found that the required concentration of synthetic sgRNA to produce a given inhibitory effect was higher than that required for sgRNA-expressing plasmids; see Figure 4. This is perhaps not surprising given that each plasmid produces multiple sgRNAs over the course of an experiment, but may also indicate some level of RNA misfolding (or possibly degradation) within the cell-free TXTL. While this protocol supplied the required sgRNA up front, the dCas9 protein was still being synthesized from a plasmid within the TXTL. Therefore, another possibility, which we did not explore in these experiments, would be to replace the dCas9-expressing plasmid with some concentration of purified dCas9 protein in the initial conditions. This might be expected to further speed up the reaction and thus shorten the overall protocol. {Overall, therefore, our results hint at displacement activity but do not conclusively demonstrate this, and the effect size we observed was modest, with up to 30% recovery of reporter fluorescence observed, though levels of around 20–30% were observed in the presence of control remover RNAs without the linear binding region. Thus, our results do not provide strong evidence either for or against the displacement reaction occurring in our system; which is consistent with previous work that attempted to use RNA strand displacement for control of CRISPR interference systems in live *E. coli*.^46^

Future work in this direction may shed further light on the mechanisms involved in this system. In particular, lengthening available binding region of the sgRNA spacer should significantly reduce the off-rate of bound CRISPR ribonucleoproteins^47^ but would be expected to have less of an effect on the rate of any strand displacement processes—this might therefore be a good experiment to provide further evidence for displacement-mediated removal. Recent work on rates of RNA strand displacement, across both RNA:RNA duplexes and RNA:DNA hybrid duplexes,^33^ could shed light on any limitations in the RNA strand displacement process; more recent work also supports this possibility.^34^ Follow-up work could explore how these trends hold for additional and to explore further the effects of the overhangs guide RNAs and/or remover RNAs on the removal process, in addition to investigating more intermediate binding region lengths between 0 and 40 nt. Other biochemical mechanisms could be involved; for example, helicase activity or other activities of enzymes present in the cell-free mixture; it is known that transcription driven by very strong promoters can eject bound Cas proteins from target DNA,^48^ for example. Future work could also explore alternative methods for removing bound CRISPR/Cas ribonucleoproteins, for example, by displacing along the DNA itself rather than the spacer of the bound guide RNA, although this would require the formation of an appropriate toehold domain within the bubble generated by the binding of the Cas protein to the target DNA, unless the guide RNA could be used to recruit the invader strand. Finally, computational structural modeling of the Cas protein and guide RNA bound to target DNA, possibly guided by crystal structures,^49^ could be used as a basis to understand the biophysics of the guide RNA displacement process, which might shed further light on the processes involved and how they might be controlled. Given the rapidly expanding practical applications of CRISPR systems in biomedicine and synthetic biology, understanding mechanisms to control their behavior is likely to be of substantial importance.

## 4 Materials and Methods

### 4.1 Chemical Reagents

*E. coli* cell-free TXTL extract was purchased from Daicel Arbor Biosciences (Ann Arbor, MI). TE buffer (100×, molecular biology grade) was obtained from Millipore Sigma (Burlington, MA); this was diluted down to 0.1× as required using nuclease-free water obtained from Sigma-Aldrich (St. Louis, MO). Golden Gate restriction enzymes and T4 ligase were purchased from New England Biolabs (Ipswich, MA). T4 ligase buffer was purchased from Promega (Madison, WI). DNA oligonucleotides, including sequencing primers, were synthesized by Integrated DNA Technologies (IDT; Coralville, IA) and resuspended in nuclease-free water based on the manufacturer’s quantitation. Synthetic single guide RNAs were synthesized as “Alt-R CRISPR Custom Guide RNAs” by IDT and resuspended similarly. Double-stranded gene fragments were synthesized by IDT and resuspended in 10 mM Tris-HCl (pH 8) at a concentration of 40 fmol μL^–1^ (40 nM) for subsequent use in Golden Gate reactions. 10-beta Competent *E. coli* cells and the pUC19 cloning vector were purchased from New England Biolabs. ZymoPURE II Plasmid Midiprep Kits were purchased from Zymo Research. See Table S1 for details of all nonoriginal plasmids used in this work, along with their provenances.

### 4.2 Plasmid Design and Assembly

Original plasmids cloned for this work are listed in Table S2; full sequence information is included in the correspondingly named GenBank data files included as additional Supporting Information. Plasmids were cloned using Golden Gate assembly techniques using established protocols^50,51^ pMRL18 and subsequently numbered plasmids were cloned using the CIDAR MoClo Golden Gate scheme by inserting double-stranded gene fragments, synthesized and prepared as outlined above. Reaction products were transformed into NEB 10-beta Competent *E. coli* cells using standard protocols and cultured, after an initial outgrowth step in NEB SOC outgrowth media (B9020S), on LB-agar with appropriate antibiotics. Plasmids were extracted using Zymo plasmid Miniprep kits and quantitated using a NanoDrop 2000c spectrophotometer (Thermo Scientific). Functional regions of novel constructs were sequence verified using Sanger sequencing by Genewiz (South Plainfield, NJ); sequencing primers used with each plasmid are listed in Table S3. Sequences for small RNAs expressed by these plasmid constructs were designed as outlined below.

### 4.3 Sequence Design for DNA Oligonucleotides and Small RNAs

Sequences for DNA oligonucleotides, remover RNAs, and extended CRISPR guide RNAs were designed using NUPACK^38,39^ along with custom Python scripts that generated candidate sequences for testing. Sequences for binding regions were chosen to minimize secondary structure and unwanted folding of the RNAs based on the NUPACK predictions and to provide the desired interactions with the deGFP reporter sequence. Results from NUPACK analyses of the secondary structures of some small RNAs used this work are presented in Figures S13–S15. Sequences of single-stranded DNA oligonucleotides used in these experiments are presented in Table S4. Sequences of guide RNAs and remover RNAs are presented in Tables S5 and S6, respectively.

### 4.4 Protocol for One-Pot Experiments

Appropriate concentrations and volume of reagents were combined in 0.2 μL PCR tubes to produce a 4× master mix for each experimental condition. Concentrations of the various chemical species used in these experiments are presented in Tables S7 and S8. As far as possible, the total concentrations of plasmid DNA across different conditions were held constant by the inclusion of “filler” plasmids that do not code for any components active in the CRISPRi system, such as pUC19, DVK_AE, or similar. (In some cases, these master mixes were prepared ahead of an experiment and frozen before being defrosted immediately prior to use.) Cell-free TXTL extract in 75 μL aliquots was stored in an ultralow temperature freezer at −80^◦^C and thawed on ice immediately prior to use and mixed by vortexing. Eighteen μL of TXTL was pipetted into each PCR tube to produce a 24 μL total volume and mixed, before being used to pipet triplicate 6 μL volumes into a Costar 3357 V-bottom 96-well plate (Corning, Salt Lake City, UT), taking care to form consistent and round bubbles in the center of the well and avoiding the formation of bubbles. The plate was sealed using Corning Costar 3080 microplate storage mats and was read for 12 h at 30^◦^C in a BioTek H1MG Synergy plate reader using bottom read, excitation and emission at 485 and 528 nm respectively, and gain set to 50. This protocol was used to generate the data for Figures 2, 3c, 4, 5, S1– S5a, and S6.

### 4.5 Protocol for Preincubation Experiments

This protocol, used for some experiments with remover-expressing plasmids, was similar to that outlined above, except that the master mixes were prepared in two parts in separate 0.2 μL PCR tubes, as follows. In the first tube, a 1.2 μL 4× master mix of the remover-expressing plasmid was created and combined with 3.6 μL of TXTL extract. For negative control conditions with no remover expressed, 1.2 μL of water was used instead. In the second tube, a 4.8 μL 4× master mix of all remaining components was created and combined with 14.4 μL of TXTL extract. Both tubes were then incubated at 30^◦^C in an Applied Biosystems ProFlex 3× 32-well PCR System for 2 h then held at 4^◦^C. As soon as possible, the tubes were combined on ice and then transferred to a V-bottom 96-well plate, then prepared and read for 12 h for fluorescence as outlined above. This protocol was used to generate the data for Figures 3d, 6, 7, S5b, S7– S12.

### 4.6 Data Analysis

Experimental data was exported as Excel files and parsed, analyzed, and plotted using custom Python scripts. Kinetic fluorescence traces were background-correcting by subtracting the average value from a TXTL-only control trace at each time point. Linear regression calculations to estimate rates of deGFP expression were carried out using the stats.linregress function from the scipy library.^52^

## Supporting Information Available

Supplementary tables containing sequences of all DNA oligonucleotides, guide RNAs, and remover RNAs used in this work; information on plasmids used in this work; and supplementary data figures (PDF)
GenBank files with annotated sequences for all novel plasmid constructs cloned as part of this work (ZIP)
