# Supplementary material for: A Study of CRISPR Ribonucleoprotein Displacement in Cell-Free Systems
Source: ACS Omega. 2025 Feb 26;10(9):9154–64. doi: 10.1021/acsomega.4c09275 (PMC11904657; doi:10.1021/acsomega.4c09275)
Supplement: Supplementary file 1 — ao4c09275_si_001.pdf [file ao4c09275_si_001.pdf]

A study of CRISPR ribonucleoprotein displacement in cell-free systems
SUPPORTING INFORMATION

Randi L. Smith[1], Peter W. Davenport[2,1], and Matthew R. Lakin[2,3]

[1] Center for Biomedical Engineering, University of New Mexico, Albuquerque, NM 87131, USA
[2] Department of Computer Science, University of New Mexico, Albuquerque, NM 87131, USA
[3] Department of Chemical & Biological Engineering, University of New Mexico, Albuquerque, NM 87131, USA

January 31, 2025

S1 Supplementary Tables

Table S1: Non-original plasmids used in this work. Short names used above for simplicity are correlated to the full original name given to each plasmid by its original producer. Plasmids with providence "V.N." were provided as a gift by Prof. Vincent Noireaux. Plasmids with providence "CIDAR" are from the CIDAR MoClo Golden Gate cloning kit [1], which was obtained from Addgene. Plasmids with providence "N.E.B." were obtained commercially from the manufacturer, New England Biolabs.

**Short name**	**Full original name**	**Description**	**From**	**Used in Figs.**	**Ref.**
p70a:deGFP	P70a-deGFP_ColE1_Amp	Expresses deGFP reporter.	V.N.	all	[2]
p_dSpyCas9	dSpyCas9-cBAD33 (pRL105)	Expresses dCas9.	V.N.	all except S2	[2]
p_sg9	pCSM180-ColE1-Amp-sgRNA-anti-P70a-pos9	Expresses sg9, an on-target anti-deGFP sgRNA.	V.N.	2(b,c)	[2]
p_sgNT	pCSM169-ColE1-Amp-sgRNA-anti-P70a-nontargeting	Expresses sgNT, an off-target control sgRNA.	V.N.	2, 3, S1, S3, S4, S5, S6	[2]
DVK_AE	DVK_AE	Empty cloning vector, used as a control plasmid.	CIDAR	7, S12	[1]
pUC19	pUC19	Empty cloning vector, used as a control plasmid.	N.E.B.	2, 3, 6, S1, S2, S3, S5, S10, S11	[3]

Table S2: Original plasmids used in this work. Full sequences for these plasmids, with annotations, can be found in the correspondingly named Genbank files in the additional Supporting Information zip file.

Name	Description	Used in Figs.
pPWD2-msMRL01	Expresses rsg9 (16 nt binding region variant), J23119 promoter, ColE1, AmpR.	2, S1, S3
pPWD3a-msMRL05	Expresses R16 remover RNA, J23119 promoter, ColE1, AmpR.	2(d,e), S3
pPWD3a-msPWD20	Expresses non-interacting control remover RNA (toehold only), J23119 promoter, ColE1, AmpR.	5, S4, S6, S7, S8
pMRL17	Expresses rsg9 (40 nt binding region variant), J23119 promoter, ColE1, AmpR.	3, S4, S5, S6
pMRL18	Expresses R40 remover RNA, J23119 promoter, pMB1*, KanR.	3, 5, 6, 7, S4, S5, S7, S8, S9, S10, S11, S12
pMRL19	Expresses R0 remover RNA (without a binding region), J23119 promoter, pMB1*, KanR.	3, 6, 7, S4, S5, S10, S11, S12
pMRL27	Expressed Ctrl non-interacting remover RNA, J23119 promoter, pMB1*, KanR.	6, S9, S10, S11
pMRL52	Expresses shortened R40 remover RNA with 6 nt truncation at 5′ end, J23119 promoter, pMB1*, KanR.	S6
pMRL53	Expresses shortened R40 remover RNA with 12 nt truncation at 5′ end, J23119 promoter, pMB1*, KanR.	S6
pMRL54	Expresses shortened R40 remover RNA with 18 nt truncation at 5′ end, J23119 promoter, pMB1*, KanR.	S6

Table S3: Sanger sequencing primers used for sequence verification of novel plasmids cloned as part of this work.

Primer name	Sequence (5′ to 3′)	Used for plasmids
onPWD610	TTTTGTGATGCTCGTCAGGG	pPWD2-msMRL01, pPWD3a-msMRL05, pPWD3a-msPWD20.
onPWD611	TGCTCACATGTTCTTTCCTGC	pMRL17.
onPWD639	CGCTGAGATAGGTGCCTCAC	pPWD2-msMRL01, pPWD3a-msMRL05, pPWD3a-msPWD20, pMRL17.
onPWD644	GTTGAAGGATCAGCTCGAGTG	pMRL18, pMRL19, pMRL27, pMRL52, pMRL53, pMRL54.
onPWD645	TACCGCCTTTGAGTGAGCTG	pMRL18, pMRL19, pMRL27, pMRL52, pMRL53, pMRL54.

Table S4: Sequences of synthetic single-stranded DNA oligonucleotides used in the experiments reported herein. Subsequences with specific functionalities (or their reverse-complements) are color-coded as follows: sg9 spacer sequence, 16 nt linear region for remover RNA binding, dCas9 binding region.

Name	Sequence (5′ to 3′)	Used in Figs.
dCtrl	CCGGTCTCTCTCCCGACCTTGATGTTTCCAGTGCGATTGAGGAC CTTCAGTGC	S2
dR16	TAAAACAGGTGAAGTTCGAGGGCGACAGGATAGATAAGAGTG	S1, S2
dR0	TAAAACAGGTGAAGTTCGAGGGCGAC	S1, S2

Table S5: Sequences of guide RNAs used in this work. For guide RNAs where a synthetic (as opposed to plasmid synthesized) version was used, the name was prefixed with "synth_" to indicate the synthetic nature of the RNA. For example, the synthetic version of "sgNT_NoTH" was referred to as "synth_sgNT_NoTH". Subsequences with specific functionalities (or their reverse-complements) are color-coded as follows: sg9 spacer sequence, sgNT spacer sequence, 40 nt linear region for remover RNA binding, 16 nt linear region for remover RNA binding, dCas9 binding region, transcriptional terminator.

**Name**	**Source(s)**	**Sequence (5′ to 3′)**	**Used in Figs.**
rsg9 (16 nt)	pPWD2-msMRL01	*CACUCUUAUCUAUCCUGUCGCCCUCGAACUUC ACCUGUUUUAGAGCUAGAAAUAGCAAGUUAAA AUAAGGCUAGUCCGUUAUCAACUUGAAAAAGU GGCACCGAGUCGGUGCUUUUUUU*	2, S1, S3
rsg9 (40 nt)	pMRL17, Synthetic	*CAUCAUCUUUCCAUCACUAUCCCUCUUCUCCC UCUCCCCUGUCGCCCUCGAACUUCACCUGUUU UAGAGCUAGAAAUAGCAAGUUAAAAUAAGGCU AGUCCGUUAUCAACUUGAAAAAGUGGCACCGA GUCGGUGCUUUUUUU*	3, 4, 5, 6, 7(a,b), S4, S5, S6, S7, S8, S9, S10, S11, S12(a,b)
sgNT_ NoTH	p_sgNT, Synthetic	*AAGCAGAUUACGUUCAAGCAGUUUUAGAGCUA GAAAUAGCAAGUUAAAAUAAGGCUAGUCCGUU AUCAACUUGAAAAAGUGGCACCGAGUCGGUGC UUUUUUU*	2, 3, 4, 5, 6, 7, S1, S3, S4, S5, S6, S7, S8, S9, S10, S11, S12
sg9_ NoTH	p_sg9, Synthetic	*GUCGCCCUCGAACUUCACCUGUUUUAGAGCUA GAAAUAGCAAGUUAAAAUAAGGCUAGUCCGUU AUCAACUUGAAAAAGUGGCACCGAGUCGGUGC UUUUUUU*	2(b,c), 6, 7(c,d), S9, S10, S11, S12(c,d)
sgScr	Synthetic	*CGUUGGAACUAUGUAGCAAAGACGUUAUUUCU UCAUGUGCGGACCGACAUUUUGGCGCUGAUUA AGAAAGACUUGAAAAAGAGUUAUGUUAGAUCC AAAAUCA*	4

Table S6: Sequences of remover RNAs used in this work. Subsequences with specific functionalities (or their reverse-complements) are color-coded as follows: sg9 spacer sequence, sgNT spacer sequence, 40 nt linear region for remover RNA binding, 16 nt linear region for remover RNA binding, dCas9 binding region, transcriptional terminator.

Name	Source	Sequence (5′ to 3′)	Used in Figs.
R16	pPWD3a-msMRL05	*UAAAACAGGUGAAGUUCGAGGGCGACAGGAUAGA UAAGAGUGCGCAAAAAACCCCGCUUCGGCGGGGU UUUUUCGC*	2(d,e), S3
R40	pMRL18	*AGGUGAAGUUCGAGGGCGACAGGGGAGAGGGAGA AGAGGGAUAGUGAUGGAAAGAUGAUGAACGCAAA AAACCCCGCUUCGGCGGGGUUUUUUCGC*	3, 5, 6, 7, S4, S5, S7, S8, S9, S10, S11, S12
R0	pMRL19	*AGGUGAAGUUCGAGGGCGACAACGCAAAAAACCC CGCUUCGGCGGGGUUUUUUCGC*	3, 6, 7, S4, S5, S10, S11, S12
Ctrl	pMRL27	*UGCUUGAACGUAAUCUGCUUCAUCAUCUUUCCAU CACUAUCCCUCUUCUCCCUCUCCCCUAACGCAAAA AACCCCGCUUCGGCGGGGUUUUUUCGC*	6, S9, S10, S11
R40-m6	pMRL52	*AGGUGAAGUUCGAGGGCGACAGGGGAGAGGGAGA AGAGGGAUAGUGAUGGAAAGAUGAUGAACGCAAA AAACCCCGCUUCGGCGGGGUUUUUUCGC*	S6
R40-m12	pMRL53	*AGUUCGAGGGCGACAGGGGAGAGGGAGAAGAGGG AUAGUGAUGGAAAGAUGAUGAACGCAAAAAACCC CGCUUCGGCGGGGUUUUUUCGC*	S6
R40-m18	pMRL54	*AGGGCGACAGGGGAGAGGGAGAAGAGGGAUAGUG AUGGAAAGAUGAUGAACGCAAAAAACCCCGCUUC GGCGGGGUUUUUUCGC*	S6

Table S7: Final concentrations of species used in experimental setups in this work (part 1 of 2).

**Figure**	**Species concentrations**
Figure 2(b,c)	[p70a:deGFP] = 4 nM; [p_dSpyCas9] = 12 nM; [p_sgNT], [p_sg9], [pPWD2-msMRL01] = 0 or 4 nM; [pUC19] = as needed to bring total plasmid concentration to 16 nM.
Figure 2(d,e)	[p70a:deGFP] = 0.4 nM; [p_dSpyCas9] = 1.2 nM; [p_sgNT], [pPWD2-msMRL01] = 0 or 0.4 nM; [pPWD3a-msMRL05] = 0 or 16 nM; [pUC19] = as needed to bring total plasmid concentration to 18 nM.
Figure 3(c), Figure S5(a)	[p70a:deGFP] = 0.4 nM; [p_dSpyCas9] = 1.2 nM; [p_sgNT], [pMRL17] = 0 or 0.1 nM; [pMRL18], [pMRL19] = 0 or 1 or 4 or 16 nM; [pUC19] = as needed to bring total plasmid concentration to 17.7 nM.
Figure 3(d), Figure S5(b)	[p70a:deGFP] = 0.4 nM; [p_dSpyCas9] = 1.2 nM; [p_sgNT], [pMRL17] = 0 or 0.1 nM; [pMRL18], [pMRL19] = 0 or 1 or 4 or 16 nM; [pUC19] = as needed to bring total plasmid concentration to 17.7 nM.
Figure 4	[p70a:deGFP] = 0.4 nM; [p_dSpyCas9] = 1.2 nM; [synth_sgScr] = 0 or 8 nM; [synth_sgNT_NoTH], [synth_rsg9_40ntTH] = 0 or 4 or 8 nM.
Figure 5	[p70a:deGFP] = 0.4 nM; [p_dSpyCas9] = 1.2 nM; [synth_sgNT_NoTH], [synth_rsg9_40ntTH] = 0 or 8 nM; [pMRL18] = 0 or 16 nM; [pPWD3a-msPWD20] = as needed to bring total plasmid concentration to 17.6 nM.
Figure 6, Figure S11	[p70a:deGFP] = 0.4 nM; [p_dSpyCas9] = 1.2 nM; [synth_sgNT_NoTH], [synth_rsg9_40ntTH], [synth_sg9_NoTH] = 0 or 8 nM; [pMRL18], [pMRL19], [pMRL27] = 0 or 16 nM; [pUC19] = as needed to bring total plasmid concentration to 17.6 nM.
Figure 7(a,b), Figure S12(a,b)	[p70a:deGFP] = 0.4 nM; [p_dSpyCas9] = 1.2 nM; [synth_sgNT_NoTH], [synth_rsg9_40ntTH] = 0 or 8 nM; [pMRL18], [pMRL19] = 0 or 1 or 4 or 16 nM; [DVK_AE] = as needed to bring total plasmid concentration to 17.6 nM.
Figure 7(c,d), Figure S12(c,d)	[p70a:deGFP] = 0.4 nM; [p_dSpyCas9] = 1.2 nM; [synth_sgNT_NoTH], [synth_sg9_NoTH] = 0 or 8 nM; [pMRL18], [pMRL19] = 0 or 1 or 4 or 16 nM; [DVK_AE] = as needed to bring total plasmid concentration to 17.6 nM.

Table S8: Final concentrations of species used in experimental setups in this work (part 2 of 2).

Figure	Species concentrations
Figure S1	[p70a:deGFP] = 4 nM; [p_dSpyCas9] = 12 nM; [p_sgNT] = 0 or 4 nM; [pPWD2-msMRL01] = 0 or 4 nM; [dR16], [dR0] = 0 or 40 µM; [pUC19] = as needed to bring total plasmid concentration to 20 nM.
Figure S2	[p70a:deGFP] = 4 nM; [dCtrl], [dR16], [dR0] = 0 or 40 µM; [pUC19] = as needed to bring total plasmid concentration to 20 nM.
Figure S3	[p70a:deGFP] = 0.4 nM; [p_dSpyCas9] = 1.2 nM; [p_sgNT], [pPWD2-msMRL01] = 0 or 0.1 nM; [pPWD3a-msMRL05] = 0 or 16 nM; [pUC19] = as needed to bring total plasmid concentration to 17.7 nM.
Figure S4	[p70a:deGFP] = 0.4 nM; [p_dSpyCas9] = 1.2 nM; [p_sgNT], [pMRL17] = 0 or 0.1 nM; [pMRL18], [pMRL19] = 0 or 1 or 4 or 16 nM; [pPWD3a-msPWD20] = as needed to bring total plasmid concentration to 17.7 nM.
Figure S6	[p70a:deGFP] = 0.4 nM; [p_dSpyCas9] = 1.2 nM; [p_sgNT], [pMRL17] = 0 or 0.1 nM; [pMRL52], [pMRL53], [pMRL54] = 0 or 16 nM; [pPWD3a-msPWD20] = as needed to bring total plasmid concentration to 17.7 nM.
Figure S7	[p70a:deGFP] = 0.4 nM; [p_dSpyCas9] = 1.2 nM; [synth_sgNT_NoTH], [synth_rsg9_40ntTH] = 0 or 8 nM; [pMRL18] = 0 or 16 nM; [pPWD3a-msPWD20] = as needed to bring total plasmid concentration to 17.6 nM.
Figure S8	[p70a:deGFP] = 0.4 nM; [p_dSpyCas9] = 1.2 nM; [synth_sgNT_NoTH], [synth_rsg9_40ntTH] = 0 or 8 nM; [pMRL18] = 0 or 16 nM; [pPWD3a-msPWD20] = as needed to bring total plasmid concentration to 17.6 nM.
Figure S9	[p70a:deGFP] = 0.4 nM; [p_dSpyCas9] = 1.2 nM; [synth_sgNT_NoTH], [synth_sg9_NoTH], [synth_rsg9_40ntTH] = 0 or 8 nM; [pMRL18], [pMRL27] = 0 or 16 nM.
Figure S10	[p70a:deGFP] = 0.4 nM; [p_dSpyCas9] = 1.2 nM; [synth_sgNT_NoTH], [synth_rsg9_40ntTH], [synth_sg9_NoTH] = 0 or 8 nM; [pMRL18], [pMRL19], [pMRL27] = 0 or 16 nM; [pUC19] = as needed to bring total plasmid concentration to 17.6 nM.

S2 Supplementary Figures

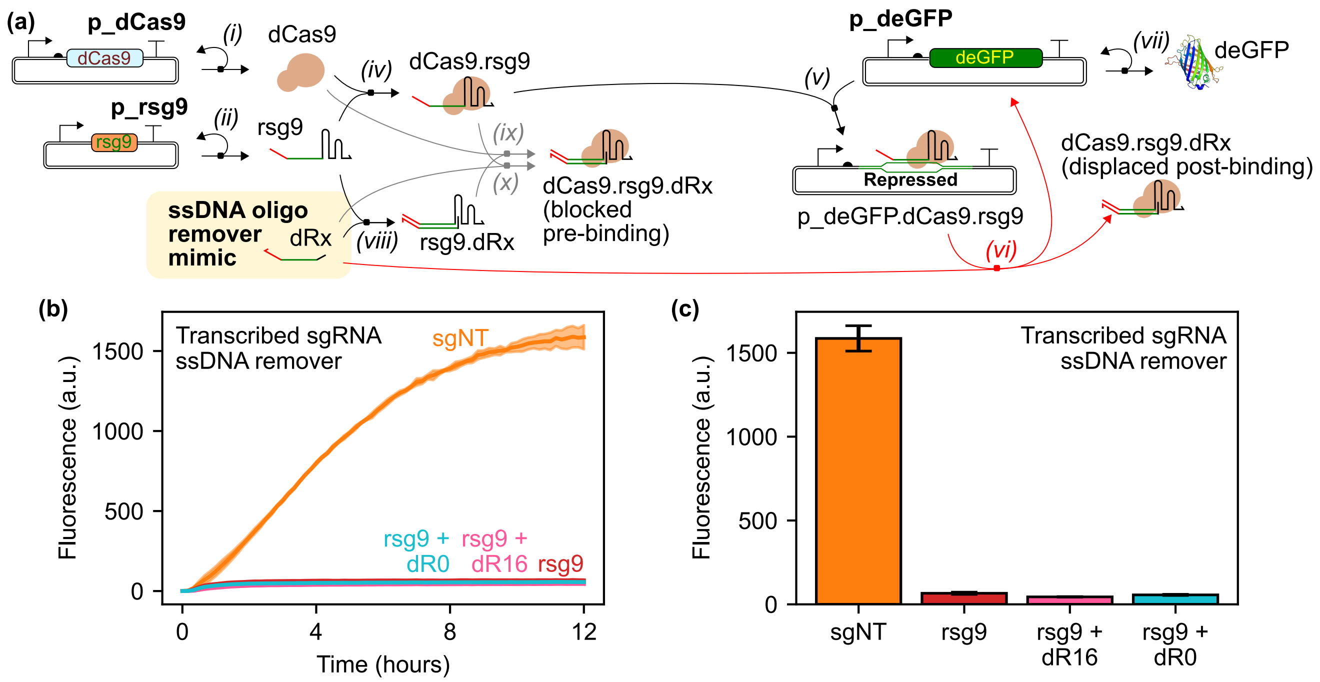

Figure S1: Initial characterization of ssDNA oligonucleotide remover mimics. **(a)** Scheme of proposed reactions in the cell-free TXTL system when ssDNA oligo nucleotide are used. The only difference is that a finite quantity of the ssDNA removers is provided at the start, rather than being synthesized continually from a plasmid during the experiment. Thus, the scheme is similar to the scheme from Figure 1(a) in the main text but with reaction *iii* removed, as the remover is no longer being synthesized from a plasmid. **(b)** Fluorescence timecourse illustrating the fluorescence signal observed when a considerable molar excess of ssDNA remover mimic (dR16) or control remover mimic with no toehold (dR0) is added to a system expressing the variant of the removable sgrna (rsg9) with a 16 nt linear binding region. Line shows mean of three replicates; shaded area shows one standard deviation. The lack of fluorescent response when the ssDNA oligos are added seems to indicate a lack of toehold-mediated removal, but could be attributed to the large (10,000-fold) excess of ssDNA interfering with the normal operation of the TXTL extract. **(c)** Endpoint fluorsecence values from the same experiment as in part **(b)**. Bars show mean of three replicates; error bars show one standard deviation.

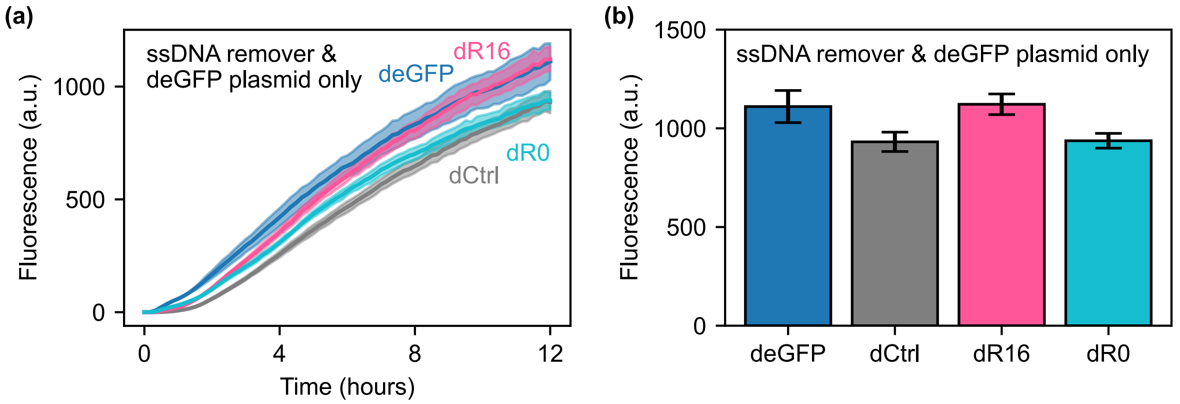

Figure S2: Experiment to characterize possible effects of large excesses of ssDNA oligonucleotide remover mimics on cell-free deGFP synthesis. **(a)** Fluorescence timecourse illustrating the fluorescence signal observed when the same large excesses of dR16, dR0 and a control ssDNA oligonucleotide (dCtrl) as in the experiment from Figure S1 are added to a cell-free TXTL reaction containing just a deGFP-expressing plasmid. Line shows mean of three replicates; shaded area shows one standard deviation. The timecourses are similar, indicating that the ssDNA itself does not seem to inhibit protein expression in the cell-free system. **(b)** Endpoint fluorsecence values from the same experiment as in part **(a)**. Bars show mean of three replicates; error bars show one standard deviation.

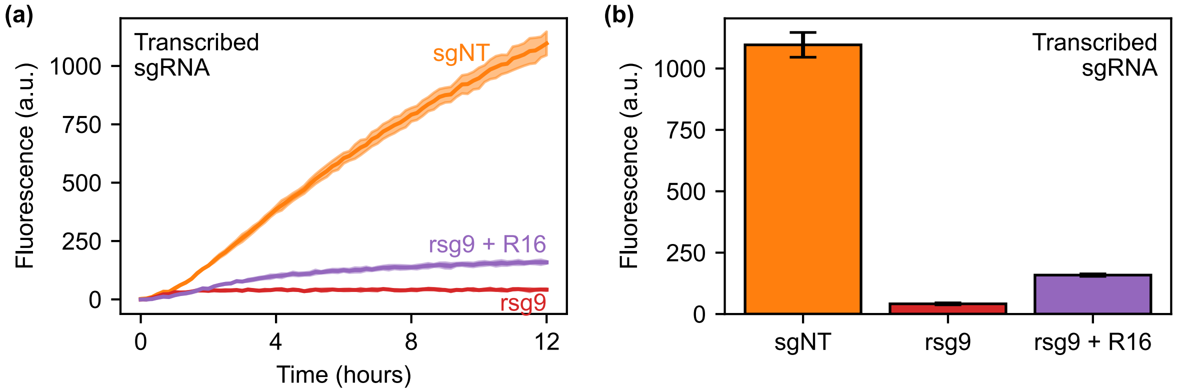

Figure S3: Additional characterization of rsg9 variant with 16 nt linear binding region and corresponding complementary R16 remover RNA. **(a)** Additional fluorescence timecourse data on removal of CRISPR RNPs by the R16 remover RNA. As in Figure 2, modest recovery of the deGFP fluorescence signal is observed. Line shows mean of three replicates; shaded area is one standard deviation above and below the mean. **(b)** Endpoint fluorescence data from the experiment shown in part **(a)**.

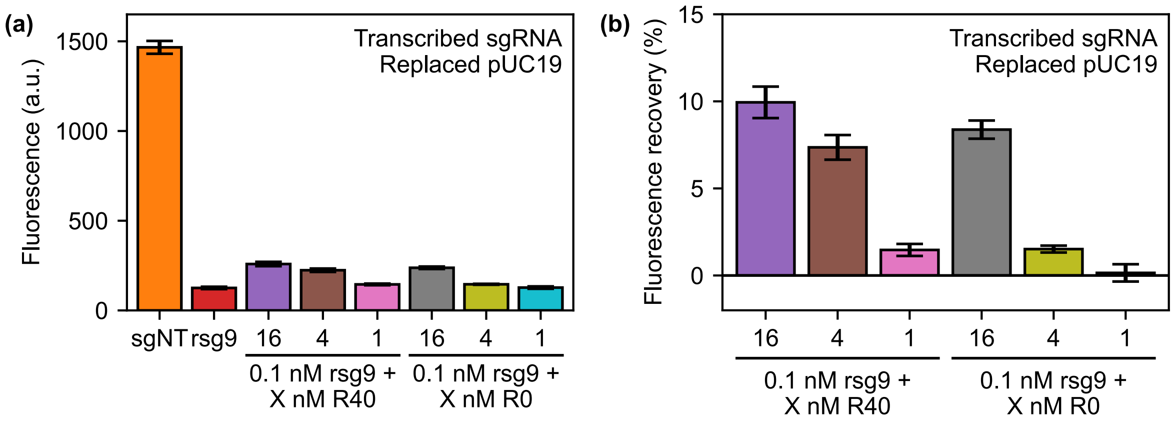

Figure S4: Experiment to determine whether the identity of the “filler” DNA plasmid, added to control for the total amount of plasmid DNA in the system, affects the behavior of the system. The experimental setup was similar to that from the experiment presented in Figure 3 in the main text, except that the commercial pUC19 plasmid was replaced with a slightly different control plasmid cloned in our laboratory (pPWD3a-msPWD20-BBaJ23119-TH05-BBa1002, see Materials & Methods for more details). **(a)** Endpoint fluorescence measurements from this experiment, with rsg9 removable sgRNA plus varying concentration of the remover-expressing plasmid. The total amount of plasmid DNA in the system was controlled with a different plasmid in this experiment, as described above, but the results were similar to those from Figure 3, indicating that the identity of the filler plasmid was not a factor in the results of that experiment. Bars indicate mean of three replicates; error bars are one standard deviation above and below the mean. **(b)** Results from part **(a)** reinterpreted as percentage recovery of fluorescence signal, interpolated between the rsg9 and sgNT controls.

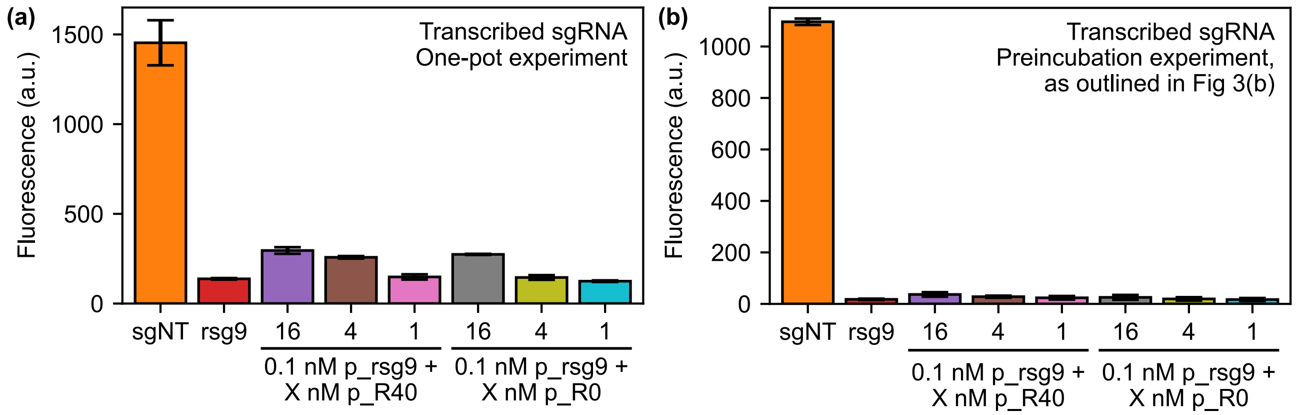

Figure S5: Unprocessed endpoint fluorescence data for the experiments presented in Figure 3 in the main text. **(a)** Unprocessed endpoint data corresponding to Figure 3(c), containing transcribed remover RNAs and transcribed removable sgRNAs in a one-pot experiment. Bars are means of three replicates; error bars are one standard deviation above and below the mean. **(b)** Unprocessed endpoint data corresponding to Figure 3(d), containing transcribed remover RNAs and transcribed removable sgRNAs in a two-part pre-incubation experiment, as outlined in Figure 3(b). Bars are means of three replicates; error bars are one standard deviation above and below the mean.

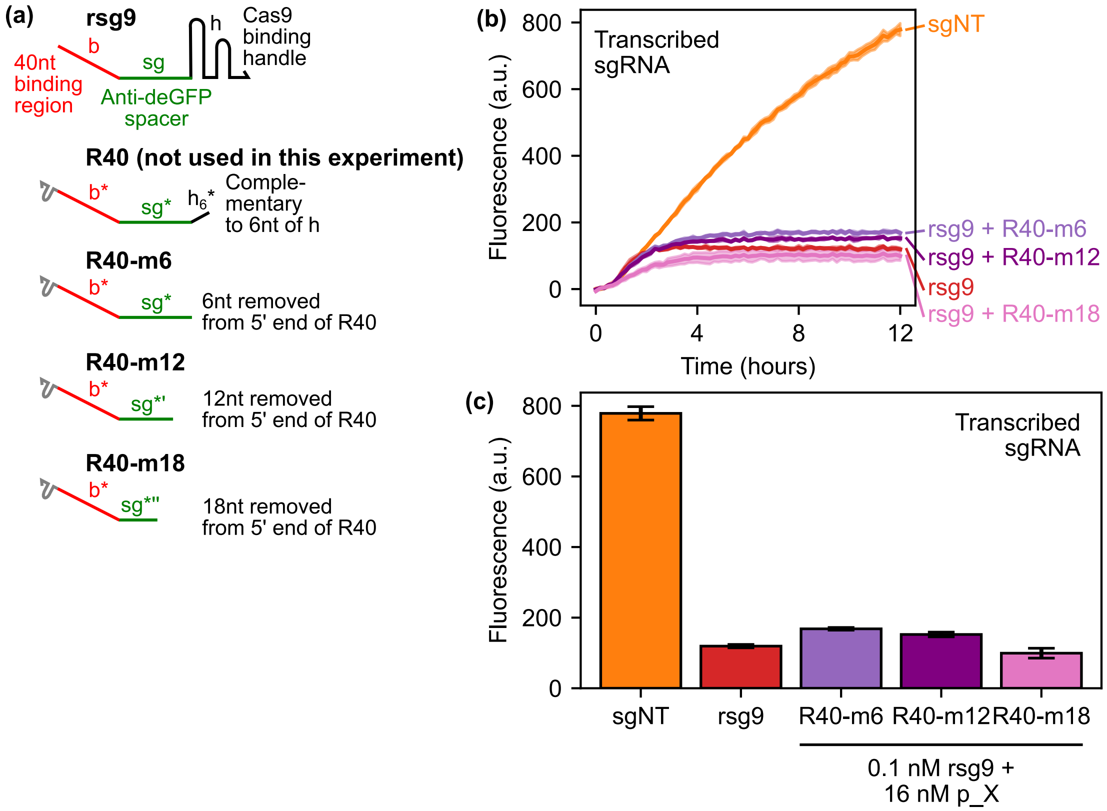

Figure S6: Characterization of truncated remover RNAs, exploring the hypothesis that a longer RNA might be sterically hindered from displacing the spacer domain by the rest of the CRISPR RNP complex, and that a shorter RNA might be able to more efficiently displace enough bases of the guide RNA from the target DNA to dislodge the complex. **(a)** Diagram outlining truncated remover RNAs used in this experiment (R40-m6, R40-m12, and R40-m18), with the original R40 remover RNA included for comparison only. Note that here we name these RNAs as truncations with respect to the full-length R40 remover, which includes 6 nt of complementarity to the Cas9 binding handle. Thus, the R40-m6 truncated remover is complementary to the whole spacer region and none of the Cas9 binding handle, the R40-m12 remover could displace all but 6 nt of the spacer, and the R40-m18 remover could displace all but 12 nt of the spacer region. **(b)** Fluorescent timecourses of the truncated removers acting on transcribed sgRNA in a one-pot experiment. Lines show mean of three replicates; shaded area is one standard deviation above and below the mean at each time point. **(c)** Endpoint fluorescence values from the same experiment as in part **(b)**. Bars represent means, error bars represent one standard deviation. We observed minimal improvement in signal due to the shortened displacement regions on these removers and thus did not use them in any subsequent experiments.

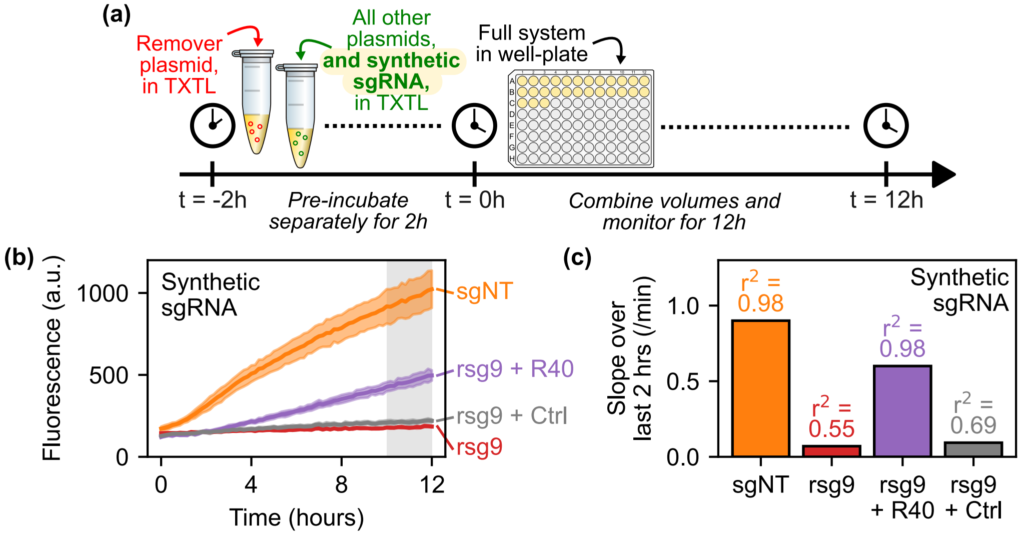

Figure S7: Studying removal of preincubated CRISPR RNPs using synthetic sgRNA. **(a)** Experiment outline for pre-incubation of CRISPR RNPs, including synthetic sgRNAs, with target DNA prior to the addition of plasmid-expressed remover RNA that has been separately incubated for the first 2 h of the experiment. (Reproduced here from main text for convenience.) **(b)** Fluorescence timecourse data showing performance of removers against pre-incubated synthetic sgRNAs. The “Ctrl” remover is a non-interacting remover control RNA. Relatively strong signal recovery was observed in the presence of the R40 remover, but not in the presence of the off-target Ctrl RNA. Line shows mean of three replicates; shaded area is one standard deviation above and below the mean. **(c)** Results of linear regression analysis over the last 2 h of the timecourse from part **(b)** (the grey shaded area in that plot). Bar indicates slope of each timecourse over that time window, annotated with the corresponding r^2 value.

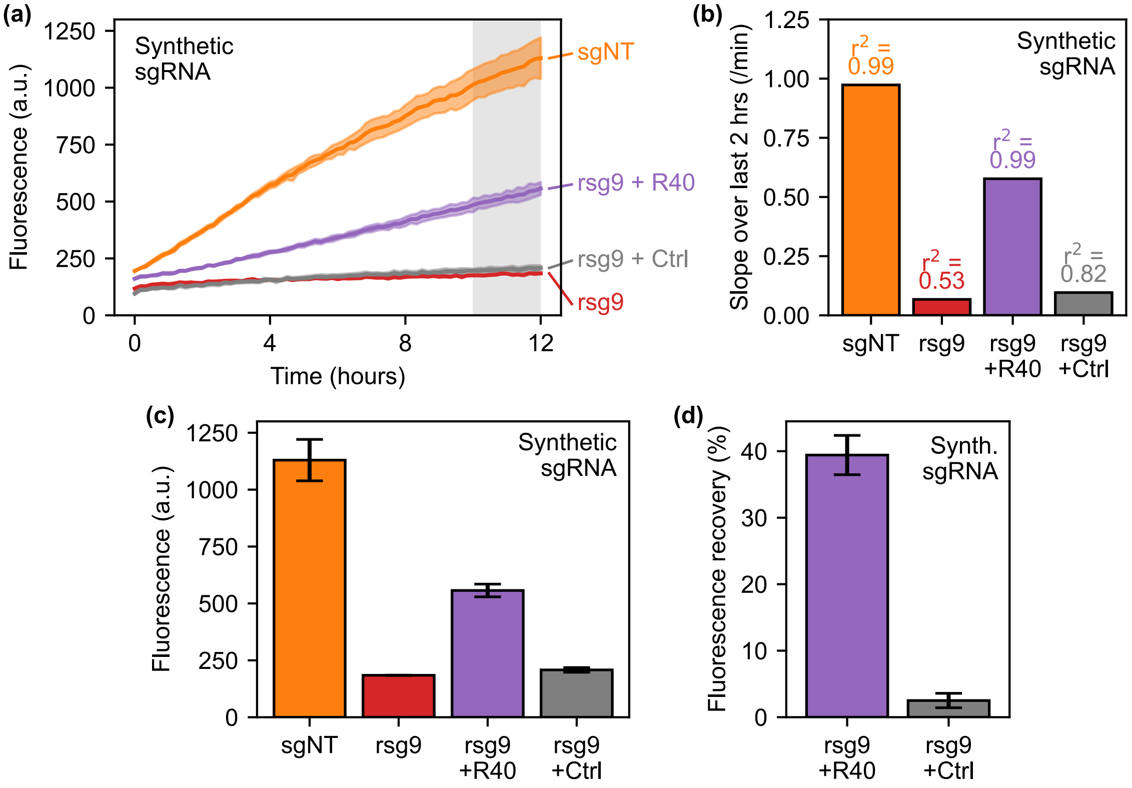

Figure S8: Results from initial replication of the R40 remover experiment presented in Figure S7. The results of this experiment support the results from that experiment, producing some evidence for antisense recovery of deGFP expression. **(a)** Fluorescence timecourse from this experiment in presence of R40 remover or scrambled control remover RNA (Ctrl). Lines show mean of three replicates; shaded area is one standard deviation above and below the mean at each time point. **(b)** Rates of deGFP synthesis in last 2 h of the experiment (grey-shaded area in part **(a)**), determined by linear regression, with associated r^2 values. **(c)** Endpoint fluorescence values from experiment in part **(a)**. Bars represent means, error bars represent one standard deviation. **(d)** Endpoint fluorescence values shown in part **(c)**., expressed as percentage fluorescence recovery interpolated between the rsg9 and sgNT controls. Bars represent means, error bars represent one standard deviation.

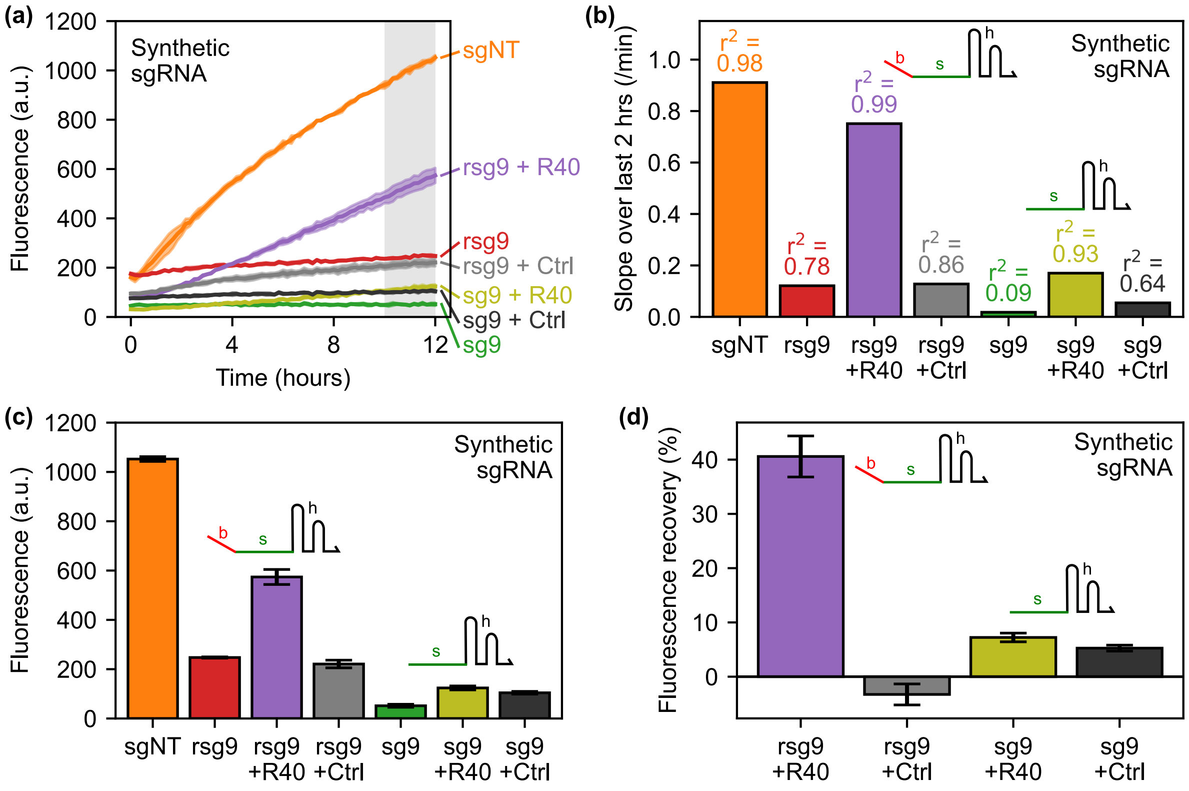

Figure S9: Results from additional replication of the R40 remover experiment presented in Figure S7. The results of this experiment support the results from that experiment, producing some evidence for antisense recovery of deGFP expression, with no such recovery seen in the presence of the scramble control remover RNA (Ctrl) or when there is no toehold on the sgRNA. **(a)** Fluorescence timecourses from this experiment when synthetic sgRNA with (rsg9) or without (sg9) a 40 nt linear binding region is used in conjunction with the R40 remover or the control RNA (Ctrl). Lines show mean of three replicates; shaded area is one standard deviation above and below the mean at each time point. **(b)** Rates of deGFP synthesis in last 2 h of the experiment (grey-shaded area in part **(a)**), determined by linear regression, with associated r^2 values. **(c)** Endpoint fluorescence values from experiment in part **(a)**. Bars represent means, error bars represent one standard deviation. **(d)** Endpoint fluorescence values shown in part **(c)**., expressed as perecentage fluorescence recovery interpolated between the rsg9 and sgNT controls. Bars represent means, error bars represent one standard deviation.

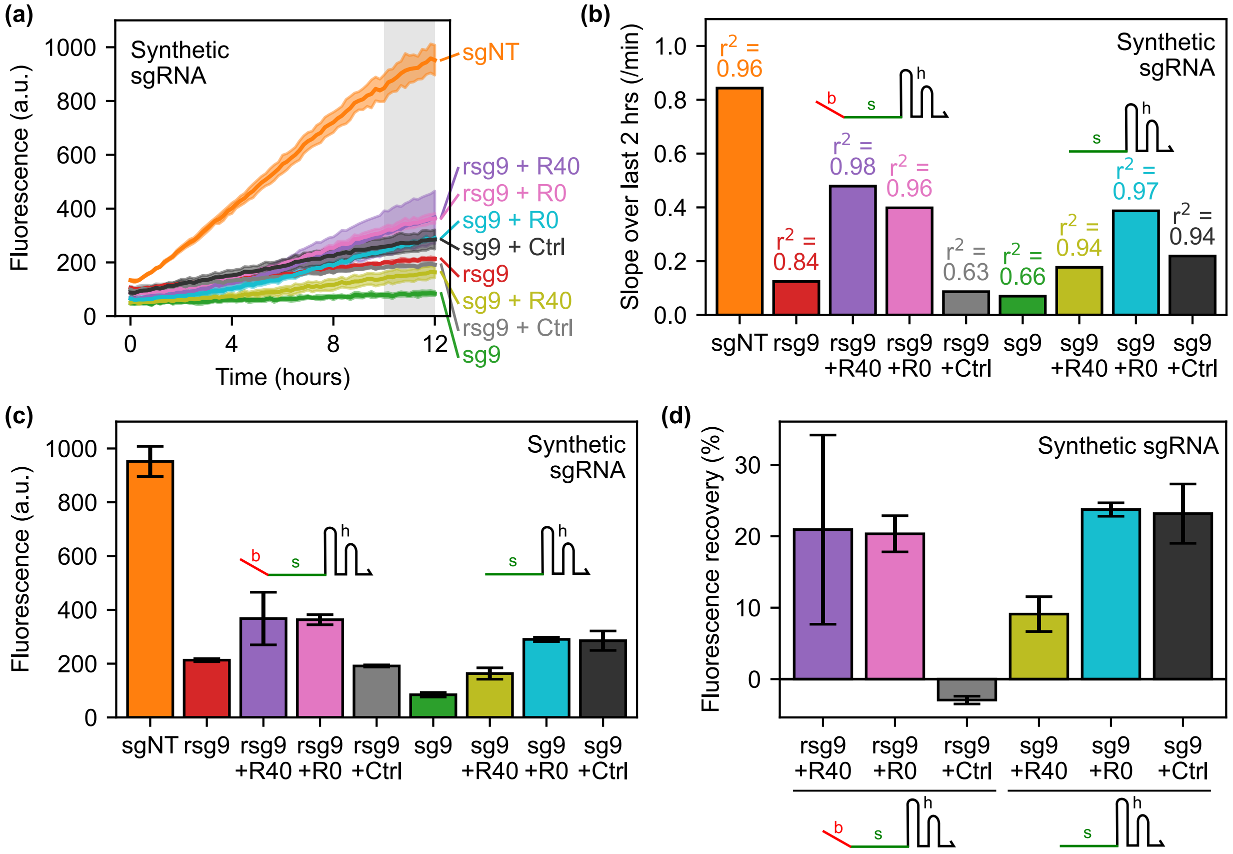

Figure S10: Results from a further experiment similar to that presented in Figure S7. The results of this experiment are more equivocal, in that relatively strong fluorescent signal recovery was seen both when the remover RNA had a linear binding region (R40) and when it did not (R0), suggesting that antisense blocking of CRISPR RNP binding may be responsible for a significant portion of the fluorescent signal observed. Interestingly, the response was weakest when the remover had a linear binding region but the sgRNA did not (sg9 + R40 condition), suggesting that the dangling unbound linear binding region on the remover RNA may destabilize the antisense interaction with the sgRNA. **(a)** Fluorescence time-courses from this experiment when synthetic sgRNA with (rsg9) or without (sg9) a 40 nt linear binding region is used in conjunction with the R40 remover, the R0 remover, or the scrambled control RNA (Ctrl). Lines show mean of three replicates; shaded area is one standard deviation above and below the mean at each time point. **(b)** Rates of deGFP synthesis in last 2 h of the experiment (grey-shaded area in part **(a)**), determined by linear regression, with associated r^2 values. **(c)** Endpoint fluorescence values from experiment in part **(a)**. Bars represent means, error bars represent one standard deviation. **(d)** Endpoint fluorescence values shown in part **(c)**, expressed as perecentage fluorescence recovery interpolated between the rsg9 or sg9 control (as appropriate) and the sgNT control. Bars represent means, error bars represent one standard deviation. Here, a negative value means a signal lower than the rsg9 or sg9 control, which could be caused by additional expression burden placed on the system.

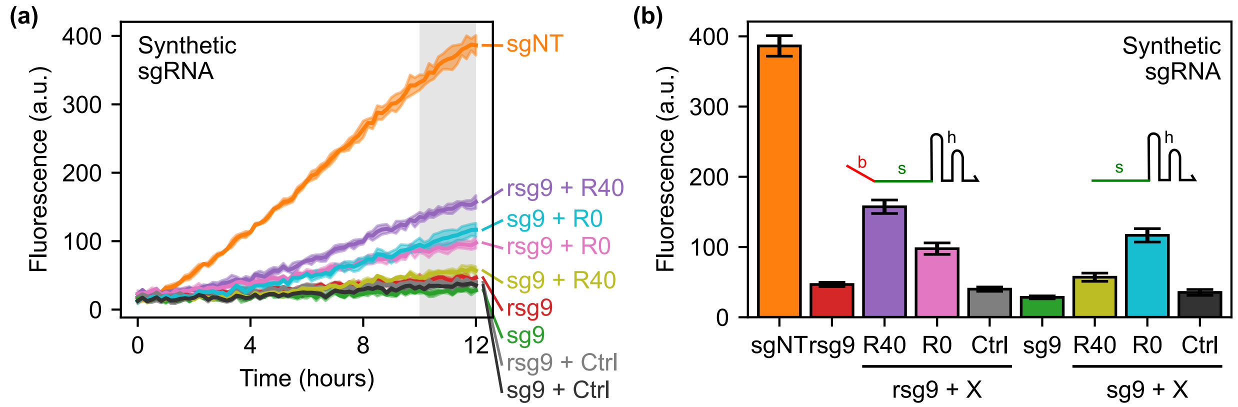

Figure S11: Raw fluoresence data used to produce the processed data presented in Figure 6 in the main text. **(a)** Fluorescence timecourses, with the 2 h time window used for the linear regression analysis in Figure 6(b) highlighted in grey. Lines show mean of three replicates; colored shaded areas are one standard deviation above and below the mean at each time point. **(b)** Endpoint fluorescence values from experiment shown in part **(a)**, which were used to calculate the percentage fluorescence recovery statistics presented in Figure 6(c). Bars represent means, error bars represent one standard deviation.

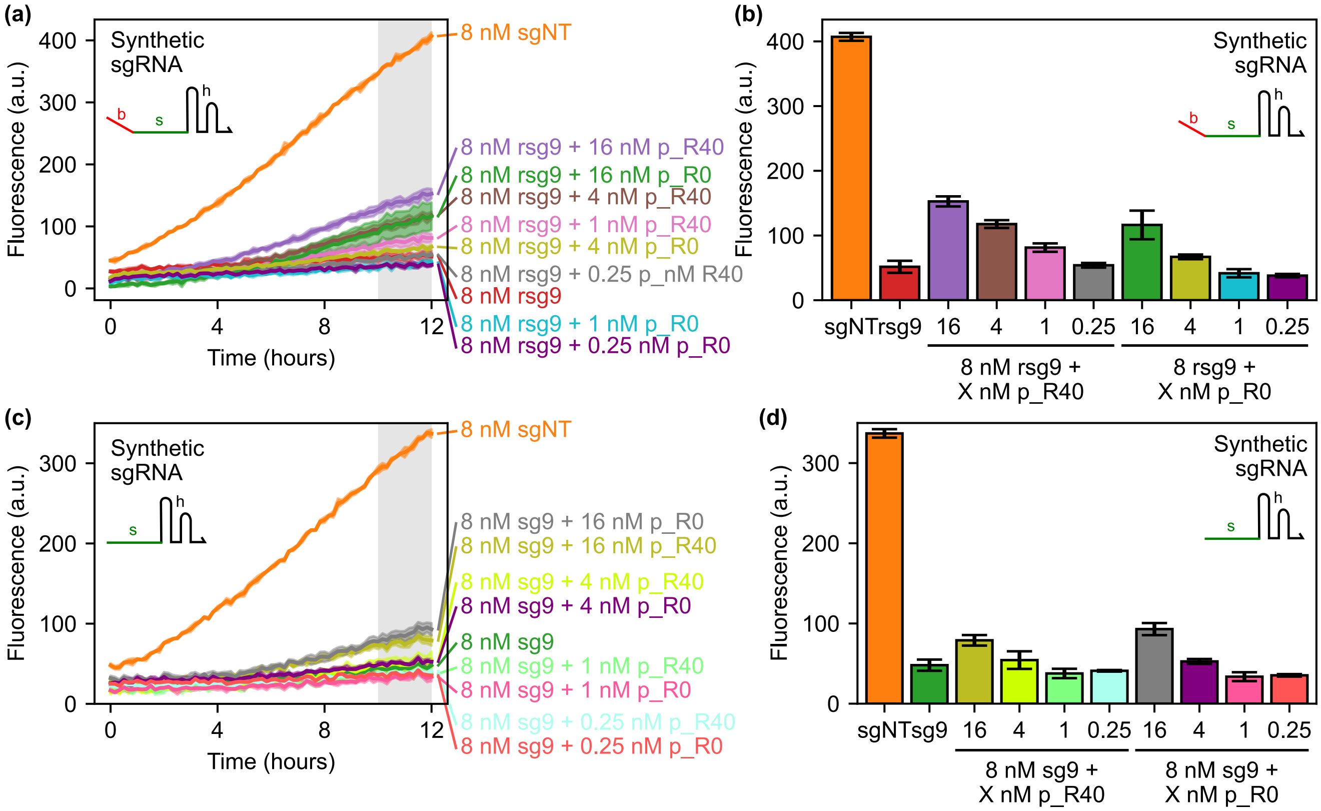

Figure S12: Raw fluoresence data used to produce the processed data presented in Figure 7 in the main text. **(a)** Fluorescence timecourses, with the 2 h time window used for the linear regression analysis presented in Figure 7(a) highlighted in grey. Lines show mean of three replicates; colored shaded areas are one standard deviation above and below the mean at each time point. **(b)** Endpoint fluorescence values from experiment shown in part **(a)**, which were used to calculate the percentage fluorescence recovery statistics presented in Figure 7(b). Bars represent means, error bars represent one standard deviation. **(c)** Fluorescence timecourses, with the 2 h time window used for the linear regression analysis presented in Figure 7(c) highlighted in grey. Lines show mean of three replicates; colored shaded areas are one standard deviation above and below the mean at each time point. **(d)** Endpoint fluorescence values from experiment shown in part **(c)**, which were used to calculate the percentage fluorescence recovery statistics presented in Figure 7(d). Bars represent means, error bars represent one standard deviation.

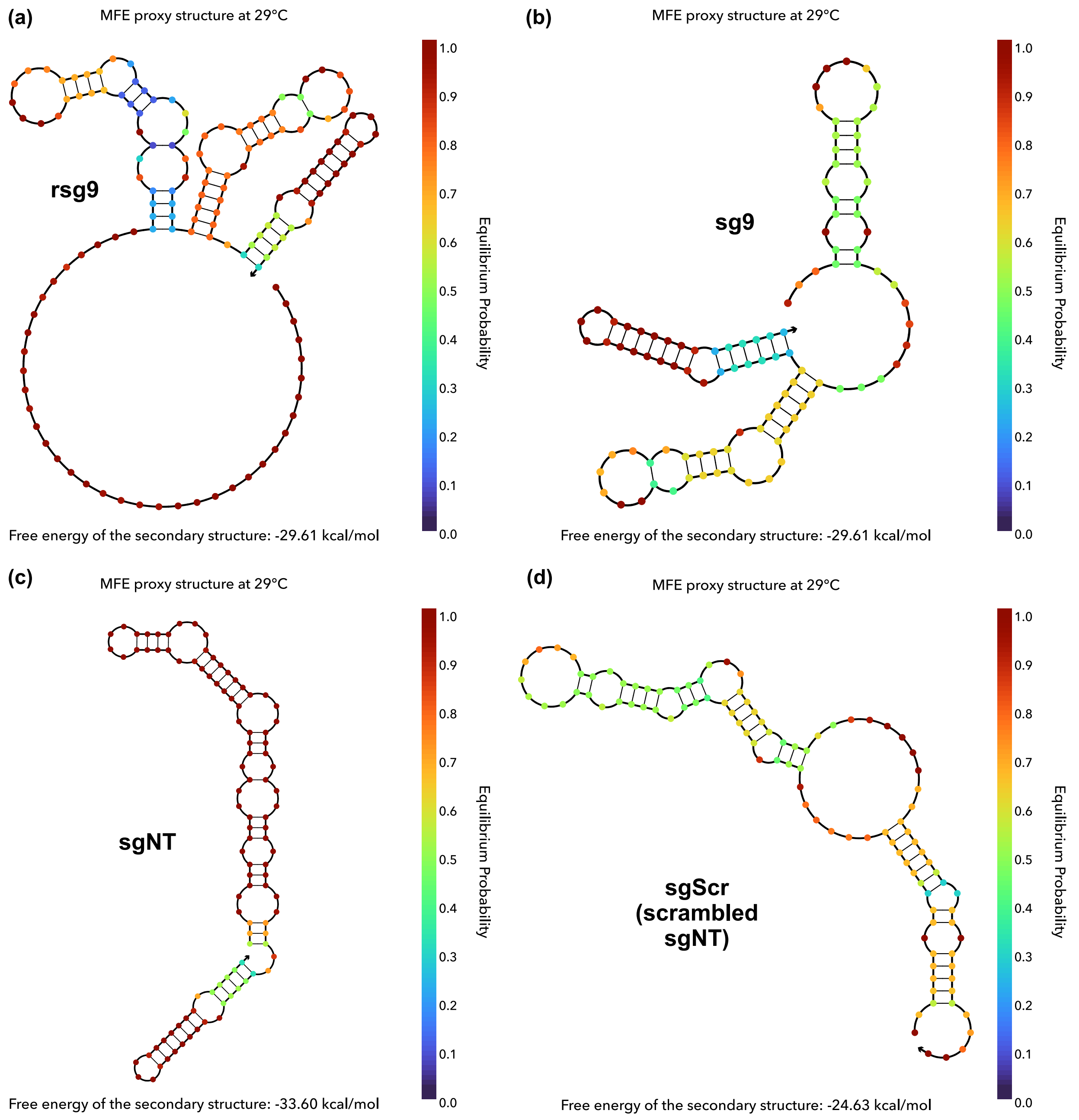

Figure S13: NUPACK prediction of minimum free energy structures for selected sgRNA sequences at 29 °C. **(a)** rsg9 removable sgRNA variant with the 40 nt linear binding region. **(b)** sg9 on-target positive control sgRNA. **(c)** sgNT off-target negative control sgRNA. **(d)** sgScr off-target negative control (scrambled version of sgNT sequence).

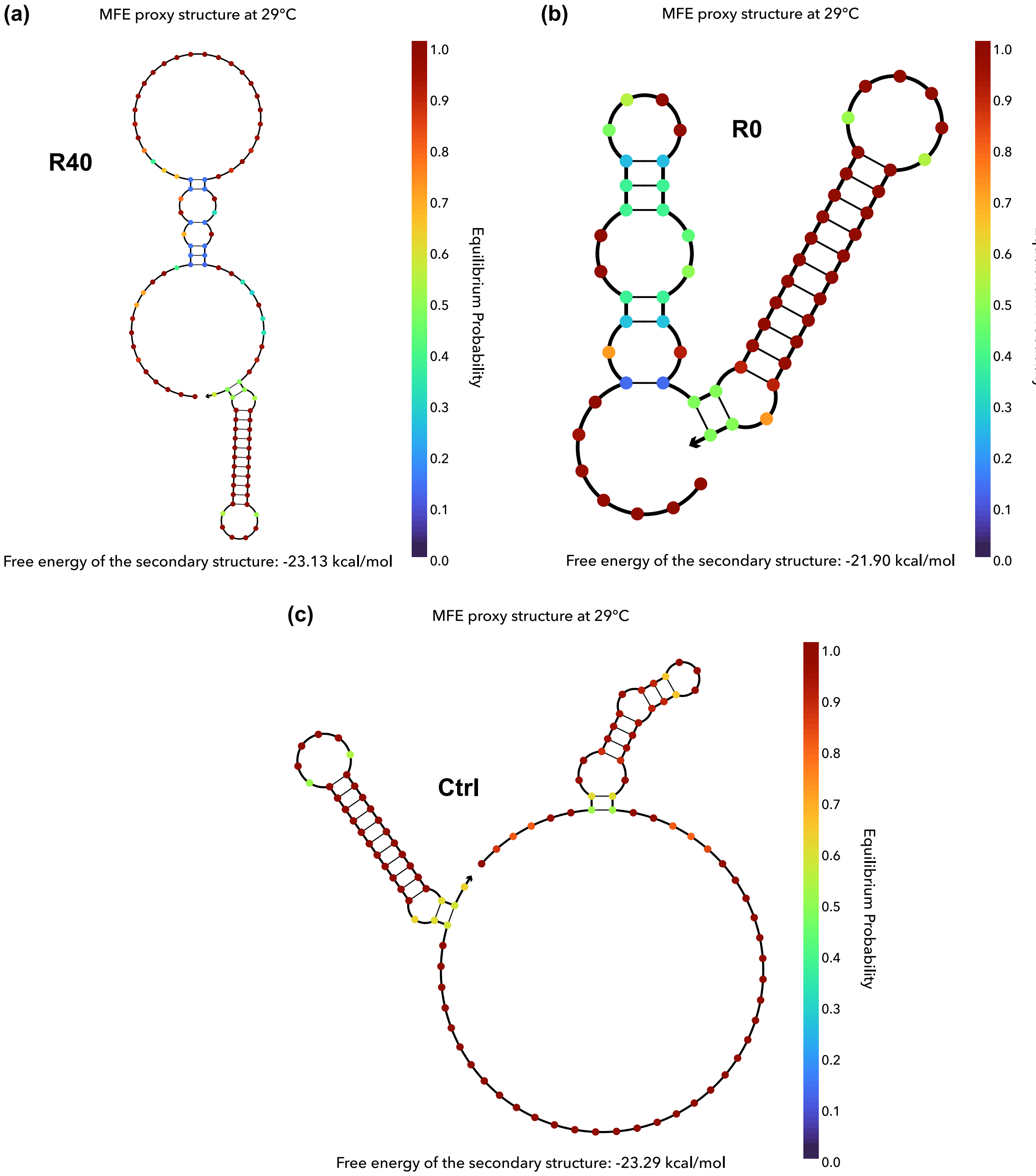

Figure S14: NUPACK prediction of minimum free energy structures for selected remover RNA sequences at 29 °C, assuming the addition of terminator hairpins with the Bba_1002 sequence. **(a)** R40 remover RNA with 40 nt linear binding region. **(b)** R40 antisense RNA with no linear binding region. **(c)** Ctrl off-target negative control RNA.

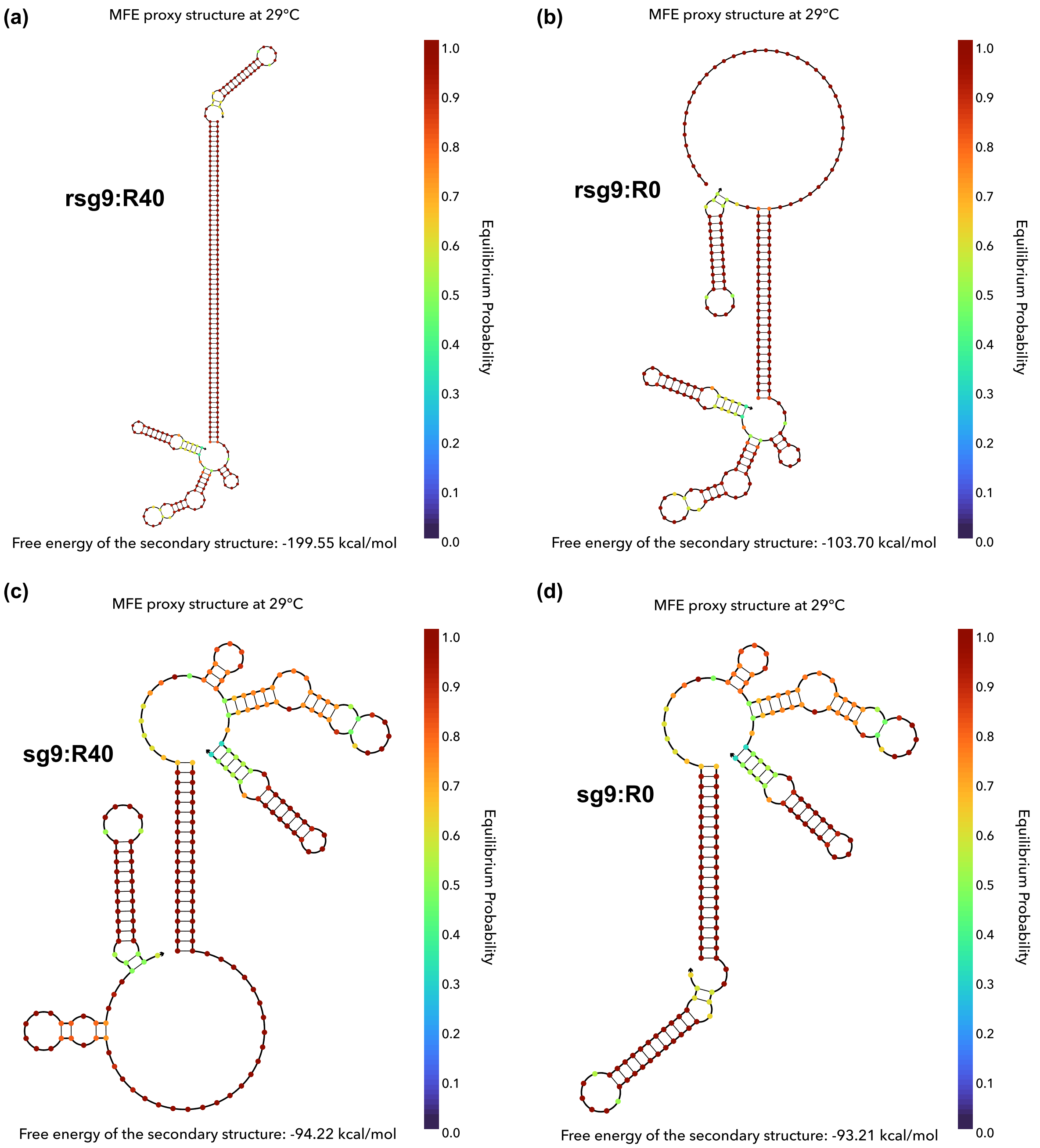

Figure S15: NUPACK prediction of minimum free energy structures for selected sgRNA:remover complexes at 29 °C, assuming the addition of terminator hairpins with the Bba_1002 sequence. Complexes were estimated for a 1 µM concentration of each individual RNA species. **(a)** rsg9:R40 complex. **(b)** rsg9:R0 complex. **(c)** sg9:R40 complex. **(d)** sg9:R0 complex.

References

[1] Iverson SV, Haddock TL, Beal J, Densmore DM. CIDAR MoClo: Improved MoClo Assembly Standard and New *E. coli* Part Library Enable Rapid Combinatorial Design for Synthetic and Traditional Biology. ACS Synthetic Biology. 2016;5(1):99-103.

[2] Marshall R, Maxwell CS, Collins SP, Jacobsen T, Luo ML, Begemann MB, et al. Rapid and scalable characterization of CRISPR technologies using an *E. coli* cell-free transcription-translation system. Molecular Cell. 2018;69(1):146-57.

[3] New England Biolabs. pUC19 Vector. New England Biolabs; 2024. Online, available from: `https://www.neb.com/products/n3041-puc19-vector`. Accessed February 14th, 2024.
